# Canagliflozin alleviates pulmonary hypertension by activating PPARγ and inhibiting its S225 phosphorylation

**DOI:** 10.1038/s41401-024-01286-9

**Published:** 2024-05-08

**Authors:** Xiu-chun Li, Xia-yan Zhu, Yang-yue Wang, Shuo-lan Tong, Zhi-li Chen, Zi-yi Lu, Jian-hao Zhang, Lan-lan Song, Xing-hong Wang, Chi Zhang, Yi-han Sun, Chu-yue Zhong, Li-huang Su, Liang-xing Wang, Xiao-ying Huang

**Affiliations:** 1 Division of Pulmonary Medicine, the First Affiliated Hospital, Wenzhou Medical University, Wenzhou Key Laboratory of Interdiscipline and Translational Medicine, Wenzhou Key Laboratory of Heart and Lung, Wenzhou, 325000 China; 2 Wenzhou Medical University, Wenzhou, 325000 China

**Keywords:** pulmonary hypertension, canagliflozin, network pharmacology, oxidative stress, post-translational modifications

## Abstract

Pulmonary hypertension (PH) is a progressive fatal disease with no cure. Canagliflozin (CANA), a novel medication for diabetes, has been found to have remarkable cardiovascular benefits. However, few studies have addressed the effect and pharmacological mechanism of CANA in the treatment of PH. Therefore, our study aimed to investigate the effect and pharmacological mechanism of CANA in treating PH. First, CANA suppressed increased pulmonary artery pressure, right ventricular hypertrophy, and vascular remodeling in both mouse and rat PH models. Network pharmacology, transcriptomics, and biological results suggested that CANA could ameliorate PH by suppressing excessive oxidative stress and pulmonary artery smooth muscle cell proliferation partially through the activation of PPARγ. Further studies demonstrated that CANA inhibited phosphorylation of PPARγ at Ser225 (a novel serine phosphorylation site in PPARγ), thereby promoting the nuclear translocation of PPARγ and increasing its ability to resist oxidative stress and proliferation. Taken together, our study not only highlighted the potential pharmacological effect of CANA on PH but also revealed that CANA-induced inhibition of PPARγ Ser225 phosphorylation increases its capacity to counteract oxidative stress and inhibits proliferation. These findings may stimulate further research and encourage future clinical trials exploring the therapeutic potential of CANA in PH treatment.

## Introduction

Pulmonary hypertension (PH) is a severe and incurable disease that seriously affects the quality of life and life expectancy of patients [1]. Although targeted drugs have been developed in recent years, they still have limitations with regard to efficacy and safety [2, 3]. Therefore, further exploration of novel medications for treating PH to improve treatment effectiveness and alleviate patient burden is urgently needed.

The primary pathological alterations in PH involve the impairment and destruction of pulmonary artery endothelial cells (PAECs), abnormal growth of pulmonary artery smooth muscle cells (PASMCs), and the accumulation of extravascular collagen [4, 5]. These pathological processes are also accompanied by the oxidative stress response of pulmonary artery cells [6]. Oxidative stress is primarily characterized by an abundance of reactive oxygen species (ROS) within cells. ROS are also critical signaling molecules for vascular cell proliferation [7, 8]. Vitry et al. showed that PH-PASMCs could exert hyperproliferative and antiapoptotic effects by hijacking persistent oxidative stress [9]. Hennigs et al. reported a PPARγ- and P53-mediated mechanism for vascular protection in PH-PAECs in response to DNA damage and oxidative stress [10]. Yeligar et al. reported that depletion of PPARγ in human PASMCs increased mitochondria-derived ROS production and disrupted mitochondrial bioenergetics [11]. These results suggest that PPARγ-driven inhibition of oxidative stress is critical for vascular remodeling in PH. Consequently, finding an effective drug targeting these pathological hallmarks is of interest for the management of PH.

Canagliflozin (CANA) is an effective hypoglycaemic drug that is approved in the USA for type II diabetes treatment. Nevertheless, in recent years, an increasing number of studies have revealed its favorable protective effects on the cardiovascular system. For example, numerous studies have indicated that CANA might impact vasorelaxation in arterioles from human visceral adipose tissue [12], hinder the proliferation of vascular smooth muscle cells in blood vessels and decrease the progression of atherosclerosis [13, 14]. Furthermore, a study revealed that CANA can substantially reduce the occurrence of major cardiovascular incidents in individuals with diabetes and chronic kidney disease [15] while also notably diminishing the cardiovascular hazard for patients suffering from heart failure [16]. The protective effect of CANA on the cardiovascular system may be attributed to its ability to inhibit cell proliferation [17], fibrosis [18], and oxidative stress [19]. However, the therapeutic effect and underlying mechanisms of CANA on PH remain unclear, and a better understanding of these effects could provide us with novel insights into PH treatment.

**Fig. 1:**
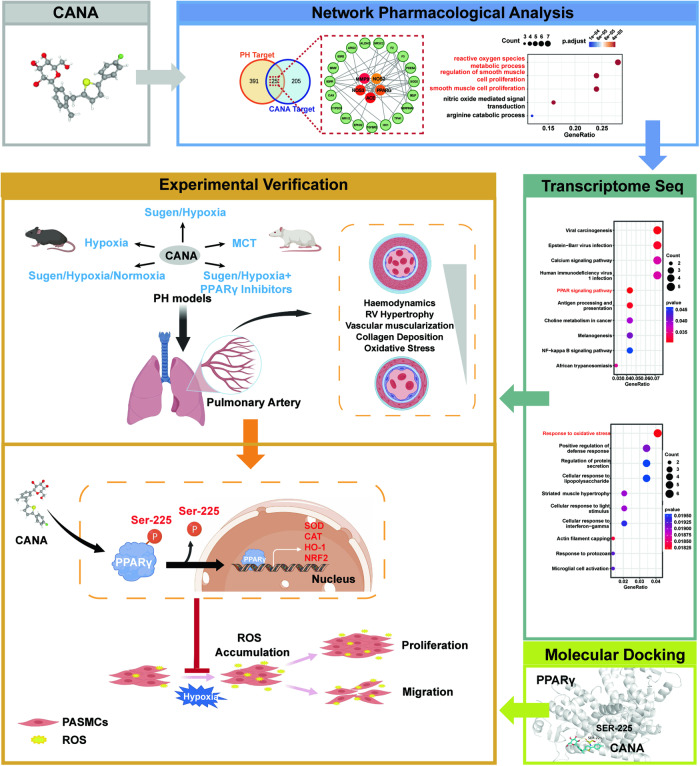
Flow chart of the study. The animation graph condensed the study method and proposed mechanism demonstrated in this work. Here, we integrate network pharmacology, transcriptomics, molecular docking, and experimental verification to uncover the role and mechanism of CANA on PH. 5 animal models are used as shown in the image. We report that CANA inhibits PPARγ Ser225 phosphorylation, promoting nuclear translocation of PPARγ, and enhancing oxidative stress-related gene translation, thus inhibiting ros accumulation, proliferation, and migration of rPASMCs.

Here, we combined network pharmacology, transcriptomics, and biological methods to evaluate the promising protective effect of CANA against PH. The pharmacological protective effect of CANA on PH was investigated in three common PH rodent models and a hypoxia-induced PH cell model in this study. Network pharmacology and transcriptomics methods revealed that PPARγ was the target and signaling molecule for the anti-PH effect of CANA, which suppressed oxidative stress and excessive proliferation of PASMCs. Subsequent validation experiments were conducted in vivo and in vitro to assess the anti-PH effects of CANA through PPARγ. Moreover, we showed that CANA targeted PPARγ and inhibited PPARγ S225 phosphorylation, thus exerting its antiproliferative and antioxidative effects on stress. Notably, this is the first time that CANA has been investigated and validated in the field of PH via multiple bioinformatics and biological methods. Figure 1 displays a schematic illustration of the investigation.

## Materials and methods

### Animal models

This study involved the creation of various animal models, including a mouse model of hypoxia-induced (Hx) PH, mouse and rat models of severe PH induced by Sugen 5416-hypoxia (Su/Hx), a model for rescuing established PH and a rat model of PH induced by monocrotaline (MCT) [4, 5, 20]. All mice and rats were purchased from Weitong Lihua Limited Company (Beijing, China). Before the experiments, all animals were allowed to acclimate to the pathogen-free animal habitat for a minimum of 1 week. Additionally, an ample supply of food (consisting of 24% protein, 15% fat, and 61% carbohydrates, sourced from Jiangsu Xietong Pharmaceutical Bioengineering Co., Ltd., China) and clear water was provided. The study strictly followed the regulations of the Animal Ethics Committee of Wenzhou Medical University and adhered to the prescribed procedures.

Regarding the mouse model, the mice (C57BL/6J, 8–12 weeks old, weighing 20–25 g) were divided into ten groups: the normoxia group (Nx group), the normoxia group treated with CANA (Nx+CANA group), the hypoxia group (Hx group), the hypoxia group treated with CANA (Hx+CANA group), the Sugen5416/hypoxia group (Su/Hx group), the Sugen5416/hypoxia group treated with CANA (Su/Hx+CANA group), the Sugen5416/hypoxia group treated with GW9662 (Su/Hx+GW9662 group) and the Sugen5416/hypoxia group treated with CANA and GW9662 (Su/Hx+CANA + GW9662 group), the Sugen5416/hypoxia/normoxia group (Su/Hx/Nx group) and the Sugen5416/hypoxia/normoxia group treated with CANA (Su/Hx/Nx+CANA group). The animals in the hypoxia group were placed inside a hypoxia chamber with an FiO_2_ of 10% for 21 days, while the animals in the normoxia group were subjected to indoor air exposure. Sugen5416 was administered subcutaneously at a dose of 20 mg/kg every week. CANA was treated orally at a dose of 10 mg·kg^-1^·d^-1^. GW9662 was injected intraperitoneally at a dose of 2 mg·kg^-^^1^·d^-1^. As for Su/Hx/Nx and Su/Hx/Nx+CANA group mice, after hypoxia exposure for 21 days, the mice were returned to normoxic conditions for an additional 2 weeks, and Su/Hx/Nx+CANA group mice accepted CANA by orally at a dose of 10 mg·kg^-1^·d^-1^ for 2 weeks. During the experiment, all the control groups received a corresponding volume of solvent.

Regarding the rat model, the rats (Sprague-Dawley, 8–12 weeks old, weighing 200–250 g) were divided into five groups in a random manner. These groups consisted of the control group (CTL group), monocrotaline (MCT group), monocrotaline treated with CANA (MCT + CANA group), Sugen5416/hypoxia group (Su/Hx group), and Sugen5416/hypoxia group treated with CANA (Su/Hx+CANA group). The animals in the hypoxia group were placed inside a hypoxia chamber with a FiO_2_ of 10% for 21 days, while the animals in the normoxia group were subjected to indoor air exposure. On the first day of modeling, the MCT treatment group mice were injected intraperitoneally by MCT at a dosage of 60 mg/kg, and the Sugen5416 treatment group mice were injected subcutaneously by Sugen5416 at a dose of 20 mg/kg. CANA was treated orally at a dose of 5 mg·kg^-1^·d^-1^ for 21 days. Control group mice received the same volume of solvent orally or injected.

CANA, GW9662, and Sugen5416 were dissolved in dimethyl sulfoxide (DMSO) and then diluted in 40% PEG300. After homogeneous mixing, 5% Tween-80 and 45% saline were added to the mixture to obtain a final DMSO concentration of 10%. MCT was configured with hydrochloric acid and normal saline in a ratio of 2:8. Blood glucose was detected by a glucometer (Accu-Chek® Performa test strips, Roche, Accu-Chek® Performa Combo, Roche, USA), and blood was collected over a fixed period of time in the morning. The first drop of tail blood was discarded after being cleaned with alcohol, and the second drop was tested.

### Invasive haemodynamic and RV hypertrophy measurements

Haemodynamic parameters were measured as previously described [4, 5, 20]. Mice or rats were administered intraperitoneal anesthesia with pentobarbital sodium (50 mg/kg) and maintained warm by utilizing heating pads. An intuitive-channel biosignal recording system (AD Instruments, AUS) was connected with a catheter, which was then inserted into the left common carotid artery and the right internal jugular vein. This analysis was used to determine the right ventricular systolic pressure (RVSP for mice), mean pulmonary artery pressure (mPAP for rats), and mean carotid arterial pressure (mCAP). Pressure transducers (PowerLab 8/35 multichannel biosignal recording system, AD Instruments, AUS) recorded and measured all pressures during end-expiration. Then, the animals were sacrificed and subjected to RV hypertrophy measurements. For determination of the weight, the right ventricle (RV) was separated from the left ventricle plus septum (LV + S). Subsequently, Fulton’s index (RV/LV + S) and the heart weight-to-body weight ratio (RV/BW) were calculated. Following the measurement of haemodynamics, the animals were euthanized, and their hearts and lungs were collected for subsequent assessment.

### Transthoracic echocardiography

Transthoracic echocardiography was performed using an ultrasound machine from Visual Sonics Vevo 3100. Mice were anaesthetized via 3% isoflurane inhalation followed by 1%–2% isoflurane inhalation to maintain anaesthetization. Then, the mice were placed supine on a heating pad (37 °C), and the fur of the chest was shaved with a chemical hair remover. In the parasternal short-axis view, pulse-wave Doppler imaging was conducted to assess pulmonary outflow. This assessment included measuring the pulmonary acceleration time (PAT), the ratio of PAT to pulmonary ejection time (PAT/PET), and the velocity time integral (PAVTI) at the aortic valve level. During end-diastole, the parasternal RV was examined from both long-axis and short-axis perspectives to measure the thickness of the RV free wall (RVFWT).

### Haematoxylin-eosin (HE) staining

After being fixed in 4% paraformaldehyde for 24 h, the lung tissues were immersed in paraffin and then sliced into 5 μm thick sections. These sections were subsequently dried, dewaxed, hydrated, and subjected to additional procedures. Pulmonary arteries were stained with haematoxylin and eosin (H&E) for morphological analysis. The wall thickness of pulmonary arteries with an external diameter ranging from 25 μm to 100 μm was determined with an optical microscope (Olympus, Japan.) For analysis of pulmonary arterial remodeling, the ratio of wall thickness (WT) to total thickness (TT, WT/TT) and the pulmonary artery wall area (WA) to total area (TA, WA/TA) were evaluated using Image-Pro Plus 9.0 software (Media Cybernetics, Silver Spring, MD, USA) with the analyzed images. The data represent at least 6 mice/rat per group. One complete lung section was taken from each mouse/rat. For each section, at least 4 images were evaluated in a blinded manner.

### Masson staining

Collagen deposition was evaluated by staining dehydrated paraffin sections with a Masson’s Trichrome Stain Kit (G1340, Solarbio, Beijing, China) according to the manufacturer’s instructions. The paraffin sections were dewaxed and hydrated, followed by incubation with Weigert’s iron haematoxylin for 5 min. After the weak acid working fluid was removed, the sections were washed with bluing solution for 5 s and distilled water for 1 min. Ponceau acid fuchsin staining buffer was added for 5 min. Next, the slides were incubated in phosphomolybdic acid solution for 2 min, dyed with aniline blue for 1 min, rinsed in weak acid solution, dehydrated, and mounted. With an optical microscope from Olympus, Japan, collagen accumulation in arteries with outer sizes ranging from 25 mm to 100 mm was observed. The level of collagen deposition was determined by calculating the ratio of the observed collagen area to the total area. The data were obtained from at least 6 mice/rat per group. One complete lung section was taken from each mouse/rat. For each section, at least 4 images were evaluated in a blinded manner.

### Immunohistochemical detection

Immunostaining of lung sections was performed to detect smooth muscle actin (α-SMA) using a mouse hypersensitivity two-step immunohistochemical detection reagent (PV-9005, Zhong Shan Jin Qiao, Beijing, China). For antigen repair, lung tissue sections were dewaxed with xylene and various concentrations of ethanol, followed by treatment with citrate antigen repair buffer (pH 6.0). After the samples were cooled to ambient temperature, reagent one was used to block inherent peroxidase activity for 10 min at room temperature; subsequently, the samples were rinsed three times with phosphate-buffered saline for 5 min each. For prevention of nonspecific binding, a solution was prepared by dissolving 0.3 g of BSA in 10 mL of PBS. The solution was then incubated for 1 h at room temperature to block the binding sites. The lung tissues were incubated with a primary antibody against α-SMA (1:100 dilution, sc-8432, Santa Cruz) and PCNA (1:50 dilution, sc-56, Santa Cruz) at 4 °C overnight without undergoing any washing. The blocking solution was also the antibody diluent buffer. The following day, the sections were returned to room temperature for 30 min. After a PBS wash and a 20-min incubation with reagent two, the reaction was amplified. Then, the cells were washed 3 times, and horseradish peroxidase-conjugated IgG (reagent three) was added for another 20 min at 37 °C. The tissue was covered with self-matched DAB (ab64238, Abcam, United Kingdom) at the appropriate times. After staining, the sections were subjected to haematoxylin staining of the nuclei for 5 min and then dehydrated, cleared, and sealed with neutral balsam (G8590, Solarbio, Beijing, China). The immunoreactivity of the lung sections was finally observed under a Nikon microscope. With ImageJ software, the wall thickness was determined as the media thickness index. A minimum of 4 arterioles were assessed in a blinded manner for every sample.

### In vitro model establishment

Rat pulmonary artery smooth muscle cells (rPASMCs) were isolated from male Sprague‒Dawley (SD) rats as previously described [21]. Then, the rPASMCs were incubated in Dulbecco’s modified Eagle’s medium (DMEM) supplemented with 10% fetal bovine serum (FBS) (Gibco, 10099–141, USA), 100 µg/mL streptomycin and 100 IU/mL penicillin (Gibco, 15140–122, USA). After the rPASMCs reached 80%–90% confluence, they were rinsed with a new solution of phosphate-buffered saline (PBS). Subsequently, the cells were digested and collected using 0.25% trypsin-EDTA (Gibco, 25200072, USA) before use and centrifuged for further passage. All the rPASMCs used were from the 4^th^-6^th^ generation. The cell confluence rate during treatment was 60%–70%. Cells in the normoxia group were seeded in cell culture plates and cultured in a humidified incubator at 37 °C and 5% CO_2_. The hypoxia treatment group was cultured at 37 °C, 5% CO_2_, and 5% O_2_ for 24 h. All experiments were repeated 6 times.

### Cell proliferation analysis

Cell proliferation was assessed using MTT solution from Biyuntian, China, a cell counting kit (CCK-8) from Dojindo (Kumamoto, Japan), and immunofluorescence staining. The rPASMCs were added to 96-well dishes at a density of 8000–10,000 cells/well and allowed to adhere for 6–8 h. CANA at different concentrations (from 0.5 μM to 20 μM) was added to each group under normoxic conditions. After 24 h of incubation, MTT reagent (5 mg/mL, 25 μL) was added to each well for an additional 4 h at 37 °C. Subsequently, the culture medium was removed, and 100 μL of dimethyl sulfoxide (Biyuntian, China) was added to dissolve the formazan crystals. For the CCK-8 assay, the cells were then exposed to 10 μL of CCK-8 solution for 4 h at 37 °C. Subsequently, the optical absorbance of each well was measured at 450 nm using a microplate reader to assess the cell proliferation capacity.

An EdU Kit (Beyotime, C0071s) was used. The rPASMCs were added to 24-well plates containing cell-crawling slides and allowed to adhere for 6–8 h. After modeling, the cells were incubated with EdU solution (diluted 1:1000) for 2 h, treated with paraformaldehyde for fixation, and permeabilized using Triton X‐100. Finally, the cells were stained with fluorescent dye and DAPI. The percentage of EdU-positive cells was determined using Photoshop software.

### Cell immunofluorescence assay

At room temperature, the rPASMCs were treated with 4% paraformaldehyde for 30 min, permeabilized with 0.1% Triton X-100, and blocked with 5% BSA for an additional 1 h. Afterwards, the cells were incubated with primary antibodies overnight at 4 °C (Supplementary Table 1). The next day, the cells were incubated with DAPI Fluoromount-G® (Southern Biotech, #0100–20, USA) after they were incubated with secondary antibodies. Fluorescence microscope images were acquired using a fluorescence microscope (Olympus, Tokyo, Japan). The number of positive cells was calculated and analyzed. PPARγ-Flag expression was confirmed using an anti-Flag antibody.

### Wound scratch assay

The rPASMCs were placed into a 12‐well plate at a density ranging from 6000 to 8000 cells per well. Pipette tips (20 μL) were used to create a denuded area across the diameter of the dish after the cells were cultured to 90% confluence. Images of the wounds were captured at 0, 12, and 24 h using the same field of view and analyzed with ImageJ software from the National Institutes of Health in Bethesda, MD. The cell migration distance was determined with this process.

### Cell transfection

All siRNAs and plasmids used in this study were synthesized by RiboBio Co., Ltd. (Guangzhou, China). Following the manufacturer’s instructions, Lipofectamine 3000 Reagent (Thermo Fisher Scientific, Inc.) was used to transfect the siRNAs and plasmids into cells that were 70% to 80% confluent. The silencing efficiency was confirmed by Western blotting. The cells were used for further experiments after 6 h of transfection. Supplementary Table 2 displays the siRNA and plasmid sequences utilized.

### Western blotting

For lysis of fresh lung tissues, RIPA buffer (Promega, Madison, WI, USA) combined with PMSF (ST507, Biyuntian, China) was used. Lung tissues in lysing matrix tubes (MP Biomedicals, CA, USA) were homogenized using standard homogenizers (MP Biomedicals, CA, USA). Next, the suspension was left on ice for 10 min and further collected. The supernatant of the cells was collected in the same manner as the tissue. The protein concentration was measured using a Pierce BCA protein assay kit (Thermo Fisher Scientific, 23250, USA). Protein samples, each containing 60 µg of protein, were separated using 10% sodium dodecyl sulfate‒polyacrylamide gel electrophoresis (SDS‒PAGE). Subsequently, the proteins were transferred to a polyvinylidene difluoride (PVDF) membrane with 0.45 µm pores (Millipore, IPVH00010, USA). The membrane was blocked in 5% skim milk for 90 min and then incubated overnight at 4 °C with the primary antibody (see Supplementary Table 1). Then, the membrane was treated with ECL Western blotting Substrate (Affinity, KF8003, USA) for blot analysis. The experiment was repeated 6 times using β-actin as the cytosolic marker. The analysis was conducted using Image Lab software (Bio-Rad).

### RNA extraction and qRT‒PCR

Total RNA was extracted from lung tissue or rPASMCs using RNAiso Plus (TaKaRa, #9109, Japan) and chloroform. One milligram of RNA was used to synthesize relative cDNA using the iScript cDNA Synthesis Kit (Bio-Rad, USA). SYBR qPCR Master Mix (Vazyme, Q712–02, China) was utilized for real-time PCR following the manufacturer’s instructions. β-actin functioned as an internal reference. The 2^-ΔΔCt^ method was used to calculate the proportional change in the identified genes. All primer sequences are listed in Supplementary Table 2.

### Measurement of lung MDA, SOD, CAT, and MPO activity

Fresh supernatant and homogenized solution from fresh lung tissue were collected in precooled saline. The manufacturer’s instructions were followed to quantify the supernatant and homogenize the solution using a microscale malondialdehyde assay kit (Nanjing Jiancheng Biological Institute, A003–2–1, China), a total superoxide dismutase assay kit (Nanjing Jiancheng Biological Institute, A001–1–1, China), a catalase assay kit (Nanjing Jiancheng Biological Institute, A007–1–1, China), and a myeloperoxidase assay kit (Nanjing Jiancheng Biological Institute, A044–1–1, China).

### Detection of ROS levels

For ROS detection in pulmonary vessels, a dihydroethidium (DHE) staining assay was performed. Briefly, fresh frozen lung sections were stained with DHE for 60 min using a frozen section ROS detection kit (Vigorous, Biotechnology, Beijing). After the samples were washed, the fluorescence images of the sections were observed under a fluorescence microscope (Olympus, Tokyo, Japan). The images were then analyzed with ImageJ to detect ROS generation.

To evaluate the generation of reactive oxygen species (ROS) in cells, we utilized the 2,7-dichlorofluorescein diacetate (DCFH-DA) probe to measure ROS levels according to the instructions provided in the kit (Nanjing Jiancheng Biological Institute, E004–1–1, China). Flow cytometry was carried out with a Cytoflex flow cytometer (Beckman Coulter, USA), and the analysis of the raw data files was performed using FlowJo software version 10.5 (Treestar, USA).

### Surface plasmon resonance (SPR)

SPR assays were performed as described previously [22]. We used Open SPRTM (Nicoya Life Sciences) to detect the interactions between PPARγ and CANA. The immobilization of the recombinant PPARγ protein (Sino Bio, 12019-H20B, 10 μg) was conducted on an NTA chip. The NTA chip was used with the highest flow rate (150 μL/min) for detection of the buffer HEPES-ET + 1% DMSO, which was then changed to 20 μL/min. The chip-forming surface was functionalized to prepare the ligand for fixing the His tag. Then, 200 μL of the dissolved PPARγ was added, and the mixture was injected for 4 min. After stabilization, high concentrations of CANA were injected. Different concentrations of CANA were mixed with buffer (0 μM, 10 μM, 20 μM, 40 μM, and 80 μM, with 0 μM serving as a negative control). Then, the mixture was loaded onto the chip surface at a flow rate of 20 μL/min for 240 s to allow protein and ligand binding. After that, natural dissociation occurred for 300 s. Notably, CANA exclusively binds only to proteins and does not interact with the chip surface. For removal of the analyte, the flow rate was increased to 150 μL/min. Finally, we used TraceDrawer (Ridgeview Instruments AB, Sweden) to analyze the results. According to the manufacturer’s instructions for Cytiva, an affinity (KD) between 10^−3^ and 10^−6^ for proteins and small molecule compounds indicates that they have strong binding.

### Network pharmacology and transcriptomics analysis

The predicted targets of CANA were obtained from Swiss Target Prediction [23], PharmMapper Server [24], and a systematic drug targeting tool (SysDT) [25]. The PH-related targets associated with PH were acquired from the DrugBank database (https://go.drugbank.com/), DisGeNET database (https://www.disgenet.org/), and OMIM database (https://omim.org/). To identify the central genes, we utilized the STRING database (https://string-db.org/) along with the Cytoscape plug-in “Cytohubba”. Finally, five hub genes (MMP9, ACE, NOS2, NOS3 and PPARG) were identified. DEGs were subjected to KEGG and GO enrichment analyses using the R software package.

RNA sequencing of lung tissue from the Su/Hx group and Su/Hx+CANA group was performed as previously described [12]. Briefly, after the quality control process, an RNA library was constructed with Illumina kits. Sequencing was performed on an Illumina HiSeq 3000 platform. We used the “edgeR” R package for differential expression analysis (*P* value < 0.05, |log2 FC | >1). The volcano plot and enrichment outcomes of KEGG and GO analyses were generated using the R package ‘ggplot2’. The sequencing data used in this manuscript can be found in the NCBI database SRA (PRJNA1054064).

### Docking and molecular dynamics simulations

The structures of PPARγ (PDB ID: 3VI8) and CANA were acquired from the RCSB PDB (https://www.rcsb.org/) and PubChem (https://pubchem.ncbi.nlm.nih.gov/). Molecular docking was performed using AutoDock Vina. Ligand docking was carried out based on the binding energy and number of hydrogen bonds. We utilized PyMOL (version 2.5.0) to visualize the docking results.

### Liquid chromatography equipment and operating conditions

CANA was identified in this research by a Waters Acquity ultra-performance liquid chromatography system (Milford, MA, USA). The completion of the liquid chromatographic (LC) separation system was achieved using an Acquity UPLC BEH C18 (2.1 mm × 50 mm, 1.7 μm) column that was equipped with precolumn protection. The gradient elution procedure was optimized by adjusting the following steps: from 0 to 0.5 min, using a 90% aqueous phase; from 0.5 to 1.0 min, gradually reducing to a 10% aqueous phase; from 1.0 to 1.4 min, maintaining at a 10% aqueous phase; from 1.4 to 1.5 min, gradually increasing to a 90% aqueous phase; and from 1.5 to 2.0 min, maintaining a 90% aqueous phase. The positively charged ions of the analyte and IS were accurately measured using an MS/MS triple quadrupole system called Xevo TQ-S (Milford, MA, USA). This system was equipped with an electrospray ionization (ESI) source and operated in positive multiple reaction monitoring (MRM) mode. The ion transitions for the IS and vericiguat were m/z 469.06 → 177.01 and m/z 427.10 → 108.97, respectively. The cone voltage and collision energy of the analyte and IS were 30 V and 20 eV, respectively. General parameters such as the desolvation temperature (DST), collision gas (CLG), desolvation gas (DG), cone gas (CNG) and capillary voltage (CPV) were also improved. The DST was set to 600 °C, and the CPV was set to 2.0 kV. The flow rates of CNG, CLG, and DG were 150 L/h, 0.15 mL/min, and 1000 L/h, respectively. The experiments were controlled, the data were acquired, and the data were processed using the Masslynx 4.1 software provided by the system (Milford, MA, USA).

### Dual-luciferase reporter assay

We constructed PPARγ active luciferase to assess the changes in the transcriptional activity of PPARγ. Lipofectamine 3000 reagent (Thermo Fisher Scientific, Inc.) was used to cotransfect the PPARγ S225A or PPARγ WT plasmid and Renilla luciferase (hRlucneo) control plasmid into 293 T cells with or without CANA treatment following the manufacturer’s instructions. After a 6 h period of transfection, the culture medium was replaced with fresh DMEM supplemented with 10% FBS. The following day, CANA was added to the medium. After 24 h, the cells were lysed at room temperature for 10 min. The relative luciferase activities were measured using the Dual-Luciferase Reporter Assay Kit (Promega) on a multimode microplate reader (SpectraMax® iD3, Molecular Devices, USA). The relative firefly luciferase activity in the reporter plasmid was adjusted to match that of Renilla luciferase.

### Statistical analyses

GraphPad Prism 9.0 was utilized for conducting the statistical analyses. The values represent the means with the standard error of the mean (SEM). All the results represent ≥ 6 independent experiments. The unpaired two‐tailed Student’s *t* test was used for 2 groups, and for multiple comparisons, ANOVA followed by the Tukey test was employed. No data were excluded from the experimental section.

## Results

### CANA prevents hypoxia (Hx)-induced mild PH and Sugen 5416/hypoxia (Su/Hx)-induced severe PH in mice

**Fig. 2:**
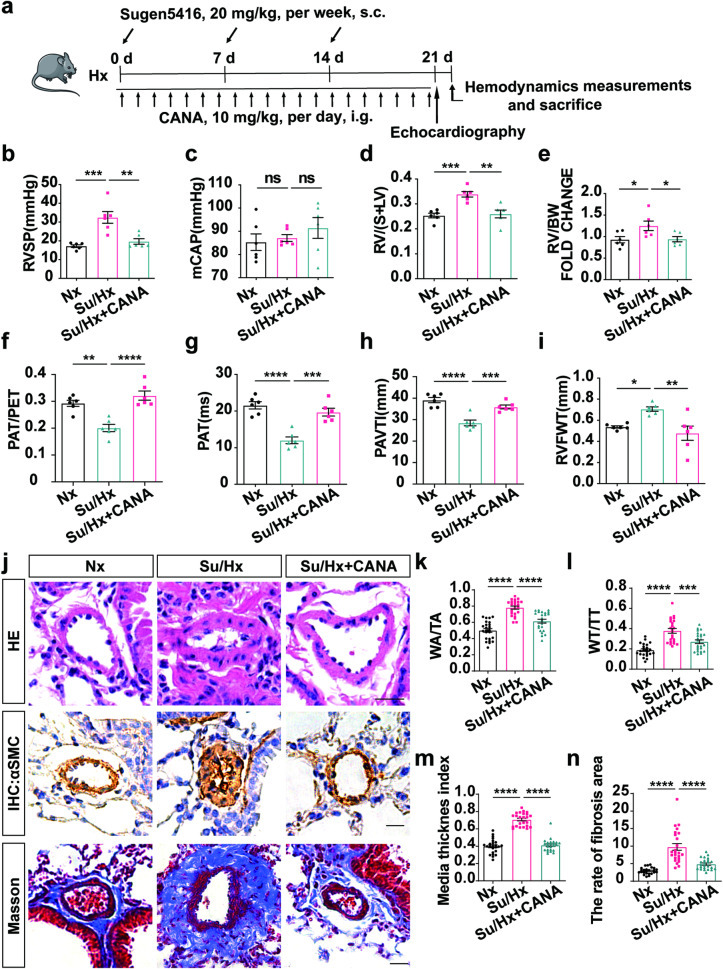
CANA alleviates Su/Hx-induced mouse PH. **a** Flowchart for Su/Hx-induced PH model establishment. **b** Quantitative analysis of the RVSP in Nx, Su/Hx and Su/Hx+CANA groups, (*n* = 6 mice per group). **c** Quantitative analysis of the mCAP in these groups (*n* = 6 mice per group). Quantitative analysis of RV/(S+LV) (**d**) and RV/WT (**e**) in these groups (*n* = 6 mice per group). Echocardiographic measurements illustrating PAT/PET (**f**), PAT (**g**), PAVTI (**h**) and RVFWT (**i**) in Nx, Su/Hx and Su/Hx+CANA groups (*n* = 6 mice per group). (**j**) Representative images of H&E staining, immunohistochemistry and Masson staining in Nx, Su/Hx and Su/Hx+CANA groups, Quantitative analysis of WA/TA (%) (**k**), WT/TT (%) (**I**), media thickness index (**m**), and the rate of fibrosis (**n**) of each group as shown in (**j**). *n* = 6 mice per group, four images per section. ^ns^*P* > 0.05, *P < 0.05, **P < 0.01, ***P < 0.001, ****P < 0.0001. Scale bars = 20 μm.

To examine the impacts of CANA, we generated a mouse model of mild PH induced by hypoxia and a mouse model of severe PH induced by Su/Hx (Supplementary Fig. S2a and Fig. 2a). The body weight of the Hx-exposed mice did not change or increased slowly due to reduced activity and foraging, whereas that of the mice under normoxic conditions increased (Supplementary Fig. S1a, c). As CANA functions as a hypoglycaemic drug, we also examined the variations in blood glucose levels in the two models. The differences in mouse blood glucose profiles among the groups were not significant, suggesting that CANA treatment under normal conditions did not cause hypoglycemic (Supplementary Fig. S1b, d). Compared with that in the Nx group, the RVSP in the Hx group and Su/Hx group increased significantly (Supplementary Fig. S2b and Fig. 2b). These results indicated that PH animal models were established successfully. After treatment with an oral gavage of CANA for 21 days, the symptoms were alleviated. CANA decreased the RVSP of PH model animals (Supplementary Fig. S2b and Fig. 2b) but had no effect on the mCAP (Fig. S2c and Fig. 2c). Further evaluation of relevant indices of right ventricular remodeling revealed that CANA markedly decreased the RV/(S + LV) and RV/BW indices, which increased in mild and severe PH (Supplementary Fig. S2d, e and Fig. 2d, e). These data suggested that right ventricular hypertrophy can return to near-normal levels after CANA treatment.

In this investigation, we analyzed PH model mice using transthoracic echocardiography. The findings indicated notable reductions in the PAT, PAT/PET ratio, and PAVTI in both PH models (Supplementary Fig. S2f–h and Fig. 2f–h). As expected, following the administration of CANA, significant increases in the aforementioned measurements were observed (Supplementary Fig. S2f–h and Fig. 2f–h). These indicators offer indirect evidence that CANA has the potential to relieve the burden on the right ventricle and reduce pressure in the pulmonary artery. In addition, we found that the RVFWT was significantly elevated in both PH models but could be restored by treatment with CANA (Supplementary Fig. S2i and Fig. 2i). Notably, no statistically significant differences in any of the abovementioned correlation indices were found between the normal group and the normal drug-treated group (Supplementary Fig. S2f–i). In conclusion, consistent with our hypothesis, CANA can alleviate exacerbating haemodynamic indicators and right ventricular (RV) remodeling in individuals with PH.

Next, we examined the effects of CANA on vascular remodeling in PH, which is characterized by pulmonary vascular muscularization and increased collagen deposition. H&E staining revealed that the pulmonary arteries in these PH models had thicker vessel walls, while CANA alleviated pulmonary artery wall thickening, specifically by decreasing WA/TA (%) and WT/TT (%) (Fig. 2k–l and Supplementary Fig. S2k, l). Furthermore, the expression of α-SMA, which is a skeletal protein of the pulmonary artery wall, was examined. Compared with that in the PH models, CANA significantly alleviated pulmonary vascular muscularization (Fig. 2k, m and Supplementary Fig. S2k, m). Moreover, the total collagen content around pulmonary vessels was significantly increased following Hx and Su/Hx treatment, and this effect was markedly reversed after CANA treatment (Fig. 2k, n and Supplementary Fig. S2k, n). Taken together, these results indicated that CANA could alleviate pulmonary vascular remodeling in mild and severe PH in mice.

### CANA prevents Su/Hx-induced and MCT-induced PH in rats

Su/Hx-induced and MCT-induced rat PH are frequently employed models for the study of PH. To further confirm the therapeutic impact of CANA on PH, we utilized these two models (Supplementary Figs. S3a and S4a). Neither MCT nor CANA treatment significantly affected the body weight or blood glucose in these rats (Supplementary Fig. S1e, f). As expected, the increased pulmonary artery pressure and worsened right ventricular hypertrophy index induced by MCT intervention or Su/Hx intervention were significantly alleviated after CANA treatment (Supplementary Figs. S3b, c and  S4b, d, e). Besides, the mCAP did not change with MCT interventions (Supplementary Fig. S4c). Transthoracic echocardiography was used to assess the effect of CANA in Su/Hx-induced PH in rats. The detection parameters of PAT, PAT/PET ratio, and PAVTI were significantly decreased in Su/Hx-induced PH rats, while they were elevated after CANA treatment (Supplementary Fig. S3d–f). Additionally, RVFWT was significantly elevated in Su/Hx-induced PH rats, while CANA reduced the Su/Hx-induced RVFWT (Supplementary Fig. S3g). As expected, Su/Hx and MCT caused pulmonary vascular muscularization (Supplementary Figs. S3h–k and  S4f–i) and increased collagen deposits (Supplementary Figs. S3l and  S4j). However, CANA markedly alleviated the deterioration of pulmonary vascular remodeling, which was reflected by the HE, α-SMA and Masson’s staining results (Supplementary Figs. S3h–l and  S4f–j). These results also suggested that CANA had a favorable therapeutic effect on PH.

### CANA reverses experimental PH in Sugen 5416/hypoxia/normoxia (Su/Hx/Nx) mice

**Fig. 3:**
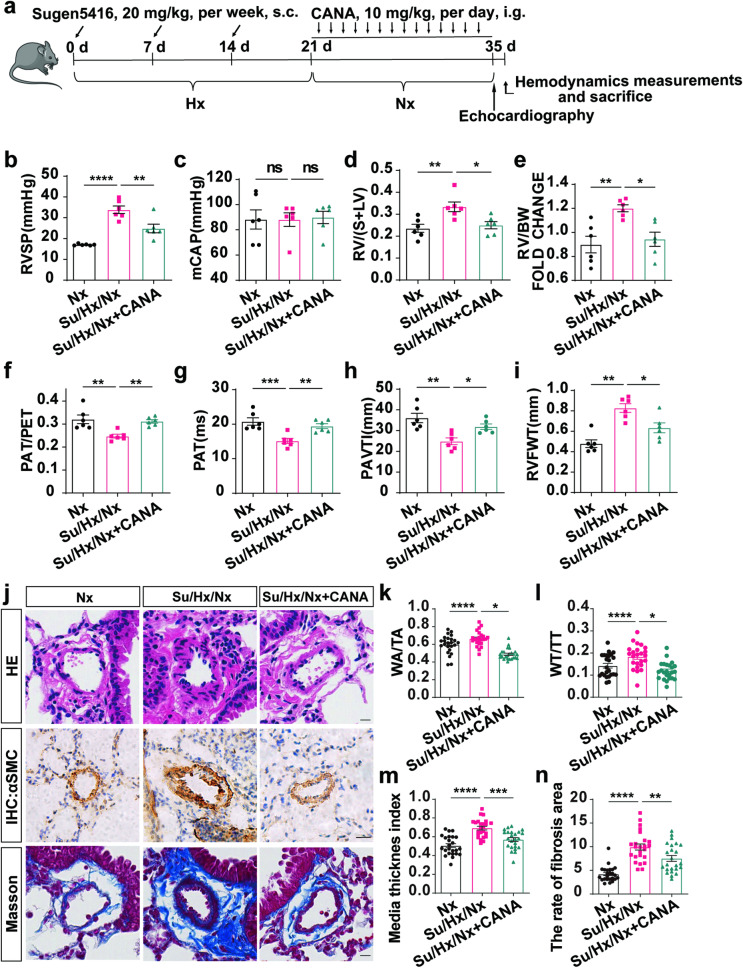
**CANA alleviates Su/Hx/Nx mouse PH.** **a** Flowchart for Su/Hx/Nx mouse PH model establishment. **b** Quantitative analysis of the RVSP in Nx, Su/Hx/Nx and Su/Hx/Nx+CANA groups. **c** Quantitative analysis of the mCAP in these groups (*n* = 6 mice per group). Quantitative analysis of RV/(S+LV) (**d**) and RV/BW (**e**) in these groups (*n* = 6 mice per group). Echocardiography measurements illustrating PAT/PET (**f**), PAT (**g**), PAVTI (**h**) and RVFWT (**i**) of these three groups (*n* = 6 mice per group). **j** Representative images of H&E staining, immunohistochemistry and Masson staining in Nx, Su/Hx/Nx and Su/Hx/Nx+CANA groups. Quantitative analysis of WA/TA (%) (**k**), WT/TT (%) (**I**), media thickness index (**m**), and the rate of fibrosis area (**n**) of each group as shown in (**j**). *n* = 6 mice per group, four images per section. ^ns^*P* > 0.05, *P < 0.05, **P < 0.01, ***P < 0.001, ****P < 0.0001. Scale bars = 20 μm.

The Su/Hx/Nx mouse model was utilized as a model for reversal. After the induction of PH, the mice were administered a daily oral dose of 10 mg/kg/d CANA or a control vehicle for 2 weeks (Fig. 3a). The mice in the Su/Hx/Nx group continued to exhibit severe PH, which was identified by disrupted blood flow patterns and the development of right-sided cardiac dysfunction. The Su/Hx/Nx group mice showed an increase in RVSP, RV/(S + LV), and RV/BW (Fig. 3b, d, e), while mCAP remained unchanged in these animals (Fig. 3c). Consistent with the haemodynamic findings, the Su/Hx/Nx mice exhibited a decreased PAT, PAT/PET ratio, and PAVTI (Fig. 3f–h), as well as a significant increase in the RVFWT (Fig. 3i). Furthermore, the Su/Hx/Nx mice exhibited increased pulmonary vascular muscularization and collagen accumulation (Fig. 3j–n).

As a rescue therapy, CANA treatment nearly reversed the changes in RVSP (Fig. 3b), RV/(S + LV) (Fig. 3d) and RV/BW (Fig. 3e); potently increased the PAT/PET ratio (Fig. 3f), PAT (Fig. 3g), and PAVIT (Fig. 3h); and reduced the RVFWT (Fig. 3i), muscularization of vessels (Fig. 3j–m), and extravascular collagen deposits (Fig. 3j, n).

### Identification of potential targets and signaling pathways of CANA in PH via network pharmacology and transcriptomics methods

To explore the potential targets and molecular mechanisms of CANA in PH, we first applied a comprehensive approach to predict the targets of CANA [23, 25, 26], and 230 drug targets were identified. Using database detection, we identified 416 PH-associated targets. A total of 25 merged targets were obtained by intersecting drug targets with PH-associated targets, and a PPI network of 25 potential targets was created (Supplementary Fig. S5a). To identify the core targets of CANA against PH, we performed a PPI network analysis based on the CytoHubba MCC algorithm of Cytoscape. Finally, five hub genes (PPARγ, MMP9, iNOS, eNOS, and ACE) were identified (Supplementary Fig. S5a). Moreover, GO enrichment analysis of the 25 CANA-anti-PH targets was performed to obtain further insight into the therapeutic mechanism of CANA in PH. The top 5 enriched genes according to the GO enrichment analysis are displayed in Supplementary Fig. S5b, indicating that CANA may target PH through important processes, such as “ROS metabolic process”, “regulation of smooth muscle cell proliferation”, and “smooth muscle cell proliferation”, which are important mechanisms through which CANA can be used for targeted treatment of PH.

**Fig. 4:**
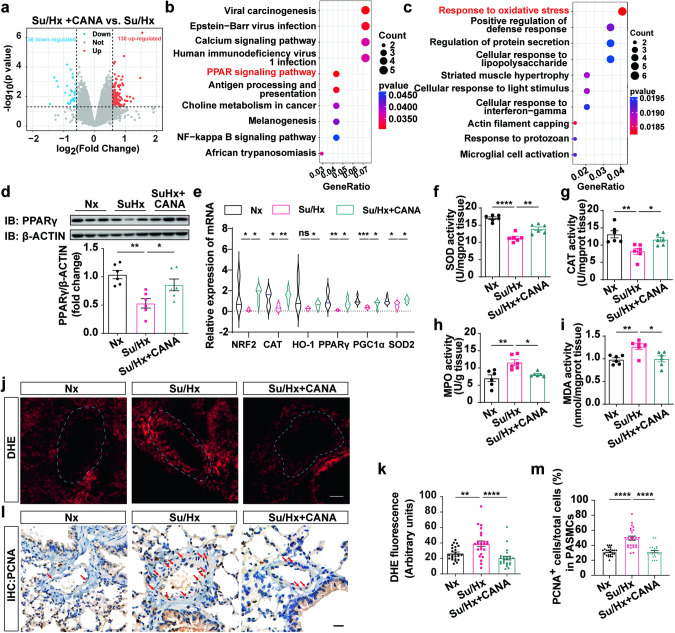
**CANA promotes PPARγ expression, and inhibits excessive oxidative stress and proliferation in vivo.** **a** Volcano plots showing the differences between Su/Hx and Su/Hx+CANA groups. **b** KEGG enrichment analysis of different expressed genes. **c** GO enrichment analysis of different expressed genes. **d** Upper panel: Representative chemiluminescence detection images detected PPARγ expression levels in lung tissue homogenates of Nx, Su/Hx and Su/Hx+CANA groups. Bottom panel: Quantitative analysis of PPARγ relative to β-ACTIN (*n* = 6 mice per group). **e** qPCR analysis of NRF2, CAT, HO-1, PPARγ, PGC1a, and SOD2 expression in mouse lung tissue of these groups (*n* = 6 mice per group). **f**–**i** Measurement of lung tissue homogenates of SOD, CAT. MPO, and MDA activity in these groups (*n* = 6 mice per group). **j** DHE staining assay was performed in fresh lung frozen sections of these groups. **k** Quantification of DHE fluorescence intensity averaged on each group (*n* = 6 mice per group, three images per section). **l** Detection of PCNA in PASMCs of Nx, Su/Hx and Su/Hx+CANA group by immunohistochemistry staining assay. **m** Quantification of PCNA^+^ cells /total PASMCs on each group (*n* = 6 mice per group, four images per section). ^ns^*P* > 0.05, *P < 0.05, **P < 0.01, ***P < 0.001, ****P < 0.0001. Scale bars = 20 μm.

To confirm the network pharmacology results, we performed transcriptome sequencing on lung tissues from the Su/Hx group and Su/Hx+CANA group mice. A volcano plot revealed significantly differentially expressed genes (Fig. 4a). KEGG pathway enrichment analysis and GO analysis were subsequently performed on the differentially expressed genes. According to the KEGG results, the “PPAR signaling pathway” was significantly enriched in the Su/Hx with CANA group compared with the Su/Hx group (Fig. 4b). Moreover, the GO results suggested that “response to oxidative stress” was significantly enriched (Fig. 4c).

Our previous research emphasized the important role of PPARγ in PH [20, 27, 28]. PPARγ is closely related to oxidative stress [29, 30], and excessive intracellular levels of ROS contribute greatly to excessive proliferation of vascular cells [7, 8]. Overall, we hypothesized that PPARγ is a target for CANA to prevent PH, which mediates the oxidative stress process in cells.

### CANA promotes PPARγ expression and inhibits excessive oxidative stress and proliferation in vivo

To verify our speculation, we determined the PPARγ protein level and characterized the effect of oxidative stress in vivo. As expected, the PPARγ expression level significantly decreased after 3 weeks of Su/Hx exposure. However, CANA promoted PPARγ expression (Fig. 4d). According to our enrichment analysis, the oxidative stress response is another important feature of CANA treatment for alleviating PH. We selected genes related to oxidative stress among the target genes of PPARγ for qPCR detection. The results revealed that the levels of antioxidant transcription factors, including NRF2 [31, 32], HO-1 [32], PPARγ [33], and PGC1α, were dramatically reduced in the Su/Hx group but increased in the Su/Hx+CANA group (Fig. 4e) [34]. Additionally, the levels of antioxidant enzymes, such as CAT and SOD, increased after CANA treatment (Fig. 4e) [35, 36]. Moreover, the enzyme activation of SOD, CAT, MPO, and MDA in fresh lung lysates was determined (Fig. 4f–i). The levels of SOD and CAT, which catalyze the dismutation of superoxide, were dramatically decreased in the hypoxia group and increased in the CANA treatment group, as expected (Fig. 4f, g). In contrast, MPO and MDA were increased in the hypoxia group and decreased in the CANA treatment group (Fig. 4h, i). Indeed, DHE staining confirmed that CANA-treated mouse lung vessels exhibited greatly reduced fluorescence (Fig. 4j, k). Additionally, the findings of immunohistochemistry indicated that CANA could significantly inhibit the Su/Hx-induced proproliferation of PASMCs in mice lung vessels (Fig. 4l, m).

### CANA inhibited oxidative stress and proliferation partially by promoting PPARγ expression in rPASMCs

**Fig. 5:**
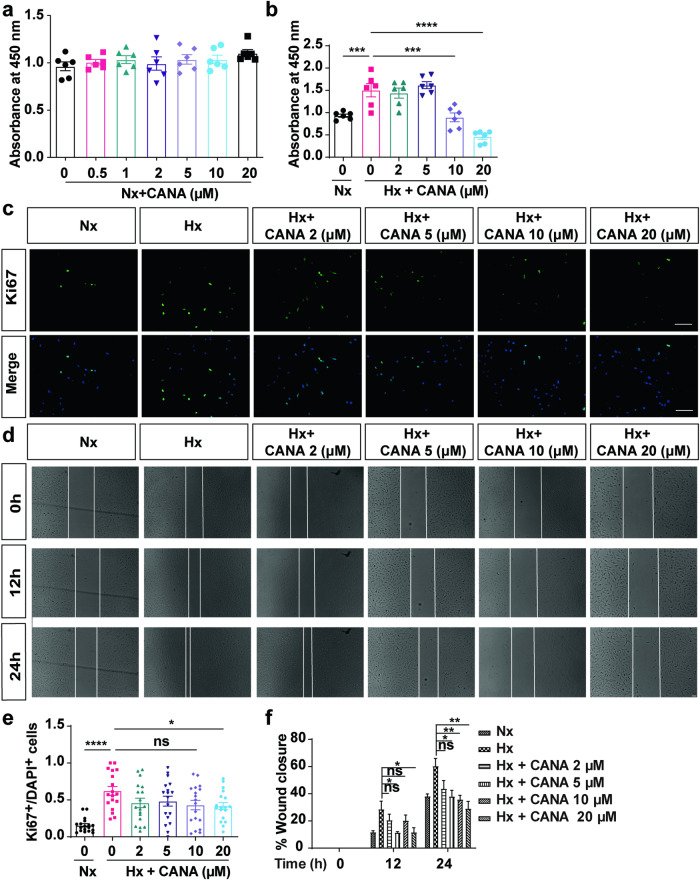
**CANA inhibits rPASMCs proliferation and migration.** **a** CCK-8 assay in rPASMCs treated with CANA (0, 0.5, 1, 2, 5, 10, and 20 μM) under normoxia condition (*n* = 6 independent experiments). **b** CCK-8 assay in rPASMCs treated with CANA (0, 2, 5, 10, and 20 μM), compared with hypoxia group (*n* = 6 independent experiments). **c** Immunostaining of Ki67 (green) in rPASMCs under different CANA concentrations (0, 2, 5,10, and 20 μM) (scale bars = 20 μm). **d** Cell migration was detected by wound-healing assays at 0, 12, and 24 h (scale bars = 100 μm). **e** Quantification of Ki67^+^/DAPI^+^ cells in each group (*n* = 6 sections from six independent experiments, three images per section). **f** Quantification of wound closure (*n* = 6 independent experiments). ^ns^*P* > 0.05, *P < 0.05, **P < 0.01, ***P < 0.001, ****P < 0.0001.

To assess the effect of CANA in vitro, we examined the effect of CANA on rPASMC proliferation and migration. CCK8 assays revealed that CANA had a weak effect on rPASMC proliferation under normoxic conditions (Fig. 5a). However, CANA at either 10 μM or 20 μM inhibited rPASMC proliferation under hypoxia (Fig. 5b). In addition, the proliferation of rPASMCs was further validated by Ki67 (a proliferation marker) immunofluorescence staining. Treatment with 20 μM CANA but not 10 μM CANA reduced the number of Ki67^+^ cells upon hypoxia exposure (Fig. 5c, e). Moreover, CANA at a concentration of 20 μM had noteworthy inhibitory effects on rPASMC migration. This inhibition was prominent at both the 12 h and 24 h time points (Fig. 5d, f). Based on these results, we chose 20 μM as the intervention concentration.

**Fig. 6:**
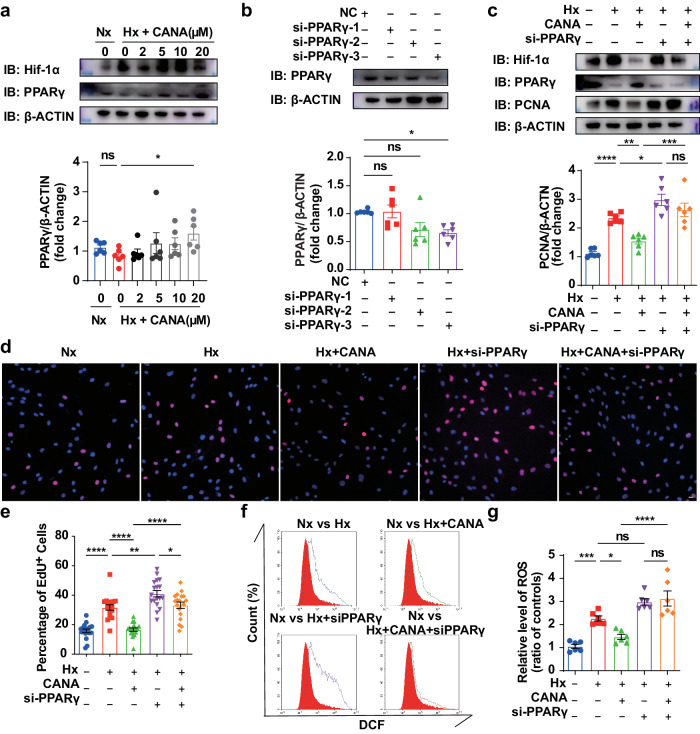
**CANA inhibits rPASMCs excessive oxidative stress and proliferation by promoting PPARγ expression.** **a** Upper panel: Chemiluminescence detection images detected PPARγ and Hif-1α expression levels in cultured rPASMCs under CANA (0, 2, 5, 10, 20 μM) treatment after hypoxia exposure for 24 h. Bottom panel: Quantitative analysis of PPARγ (*n* = 6 independent experiments). **b** Upper panel: Chemiluminescence detected PPARγ expression levels in rPASMCs transfected with three different siRNA, compared with NC group. Bottom panel: Quantitative analysis of PPARγ (*n* = 6 independent experiments). **c** Upper panel: Chemiluminescence detected PCNA, PPARγ and Hif-1α expression levels in rPASMCs treated with CANA and with or without transfected PPARγ siRNA. Bottom panel: Quantitative analysis of PPARγ (*n* = 6 independent experiments). **d** EdU staining in rPASMCs of each group. **e** Quantitative analysis of EdU^+^ cells in each group (*n* = 6 sections from six independent experiments, three images per section). **f** Intracellular ROS reacted with DCFH-DA and highly fluorescent DCF was detected by flow cytometry. **g** Quantification of DCF fluorescence on cells of each group (*n* = 6 per group from six independent experiments). ^ns^*P* > 0.05, *P < 0.05, **P < 0.01, ***P < 0.001, ****P < 0.0001. Scale bars = 20 μm.

Next, we sought to validate the results from our network pharmacology analysis and transcriptome analysis in vitro. Consistent with previous reports [20, 27, 28], PPARγ was downregulated under hypoxia, while Hif-1α was upregulated after hypoxia exposure (Fig. 6a and Supplementary Fig. S6a). In hypoxia-induced rPASMCs, CANA increased the expression of PPARγ in a concentration-dependent manner (Fig. 6a). To verify whether CANA affects cell oxidative stress and proliferation through PPARγ, we used a loss-of-function approach to knock down PPARγ expression in rPASMCs, and the results were further confirmed by Western blotting. We subsequently selected the siRNA with the most efficacious silencing effect (Fig. 6b). During PH, PPARγ plays a protective role in cell proliferation and oxidative stress [33]. As expected, PPARγ was successfully silenced by siRNA, and Hif-1α was induced by hypoxia (Fig. 6c and Supplementary Fig. S6b). As expected, silencing PPARγ induced a significant increase in PCNA expression and the proportion of EdU^+^ cells compared with those in unsilenced hypoxic cells (Fig. 6c–e). Administration of CANA partially reversed the increase in cell proliferation induced by hypoxia. However, when both molecules were coincubated, the silencing of PPARγ partially abolished the inhibitory effect of CANA on rPASMC proliferation (Fig. 6c–e). These results implied that the antiproliferative effect of CANA was partially dependent on PPARγ expression. Then, ROS levels were detected by flow cytometry to further verify the functional mechanism of CANA in PH. As expected, CANA significantly decreased hypoxia-induced ROS accumulation, while PPARγ silencing markedly abrogated the effect of CANA on ROS accumulation in rPASMCs (Fig. 6f, g). This finding suggested that knockdown of PPARγ might have a more notable effect on the antioxidant stress function of CANA than on its effect on proliferation.

In general, these findings indicate that PPARγ plays a vital role in the anti-PH mechanism of CANA, primarily by suppressing the growth and ROS accumulation of rPASMCs.

### CANA attenuates Su/Hx-induced PH partially by regulating PPARγ expression in vivo

**Fig. 7:**
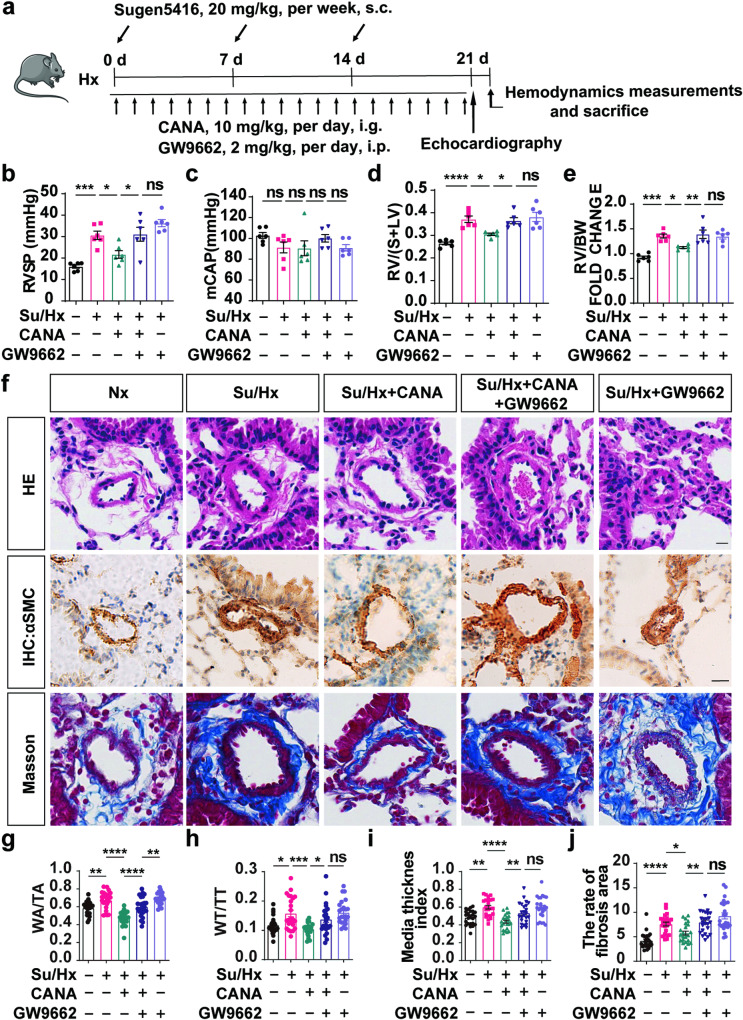
**CANA attenuates Su/Hx-induced PH partially by regulating PPARγ expression in vivo.** **a** Flowchart for the rescue mouse PH model establishment. **b** Quantitative analysis of the RVSP in Nx, Su/Hx, Su/Hx + CANA, Su/Hx + CANA + GW9662, and Su/Hx + GW9662 groups. **c** Quantitative analysis of the mCAP in these groups (*n* = 6 mice per group). Quantitative analysis of RV/(S+LV) (**d**) and RV/BW (**e**) in these groups (*n* = 6 mice per group). **f** Representative images of H&E staining, immunohistochemistry and Masson staining in these five groups. Quantitative analysis of WA/TA(%) (**g**), WT/TT (%) (**h**), media thickness index (**i**), and the rate of fibrosis area (**j**) of each group as shown in (**f**). *n* = 6 mice per group, four images per section. ^ns^*P* > 0.05, *P < 0.05, **P < 0.01, ***P < 0.001, ****P < 0.0001.

For determination of the impact of PPARγ on CANA in vivo, mice were subjected to Su/Hx for 3 weeks and administered CANA, GW9662 (an antagonist of PPARγ), or a combination of CANA and GW9662 (Fig. 7a). As expected, CANA decreased the elevated RVSP in the Su/Hx PH mice, while GW9662 worsened the SuHx-induced increase in RVSP (Fig. 7b). Moreover, the inhibition of PPARγ abolished the CANA-mediated reduction of RVSP (Fig. 7b). Moreover, there were no notable disparities in the mCAP under these conditions (Fig. 7c). In addition, the RV/(S + LV) and RV/BW of the Su/Hx-induced PH model mice clearly increased, while CANA effectively decreased this increase in the PH model mice, consistent with our previous observations. In addition, the combined use of GW9662 reversed this beneficial effect of CANA (Fig. 7d, e). As expected, CANA prominently inhibited Su/Hx-induced muscularization of vessels and extravascular collagen deposits, while the administration of GW9622 to these mice abolished the beneficial effect of CANA, further confirming that CANA alleviates Su/Hx-induced pulmonary vascular remodeling by modulating PPARγ (Fig. 7f–j).

### CANA directly binds to PPARγ and suppresses PPARγ S225 phosphorylation

**Fig. 8:**
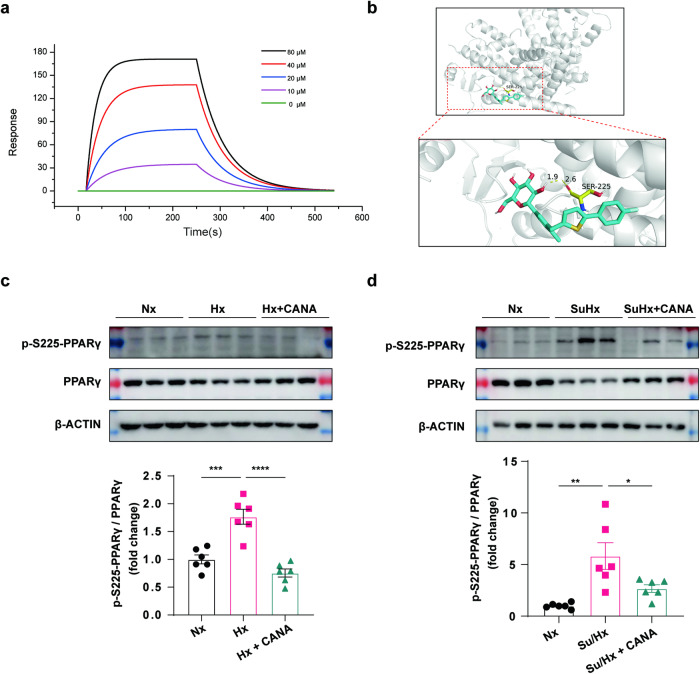
**CANA directly binds to PPARγ, inhibits PPARγ S225 phosphorylation.** **a** SPR measurements of the CANA-PPARγ interaction. **b** Results of molecular docking simulations. **c** Upper panel: Representative chemiluminescence detection images detected PPARγ expression levels in mouse lung tissue of Nx, Hx, and Hx+CANA groups. Bottom panel: Quantitative analysis of PPARγ relative to β-ACTIN (*n* = 6 per group from six independent experiments). (**d**) Upper panel: Representative chemiluminescence detection images detected PPARγ expression levels in mouse lung tissue of Nx, Su/Hx and Su/Hx+CANA groups. Bottom panel: Quantitative analysis of PPARγ relative to β-ACTIN (*n* = 6 per group from six independent experiments). *P < 0.05, **P < 0.01, ***P < 0.001, ****P < 0.0001.

The above results indicated that PPARγ might be a target of CANA. SPR was subsequently used to assess the binding of CANA in the presence of PPARγ [37, 38]. The SPR results indicated that the PPARγ protein captured on the NTA chip can bind CANA with an affinity constant of 47.6 μM (Fig. 8a).

We then performed molecular docking simulations on PPARγ (PDB: 3VI8) and CANA (DB08907). The free energy of binding between CANA and PPARγ was −6.58 kcal/mol, and the inhibition constant was 15.03 μM. The binding results revealed that CANA exhibited excellent binding activity with PPARγ (Fig. 8b). By molecular simulation docking, CANA was predicted to interact with PPARγ via Ser225 (S225, Ser225 in PPARγ1 is the same as Ser253 in PPARγ2) (Fig. 8b). Phosphorylation on serine residues was most common. Phosphorylation is the most extensive and important form of protein post-translational modification [39]. To clarify the existence of phosphorylation at the S225 site, we customized PPARγ S225 phosphorylation at HuaAn Biotechnology. Surprisingly, we found that PPAR S225 phosphorylation was significantly increased in both the Hx and Su/Hx PH models; however, CANA treatment significantly reduced PPAR S225 phosphorylation (Fig. 8c, d). Thus, we deduced that CANA competitively binds to PPARγ, which inhibits PPARγ S225 phosphorylation.

### CANA alleviates PPARγ-mediated oxidative stress and proliferative inhibition partially by preventing S225 phosphorylation and promoting the nuclear translocation of PPARγ

**Fig. 9:**
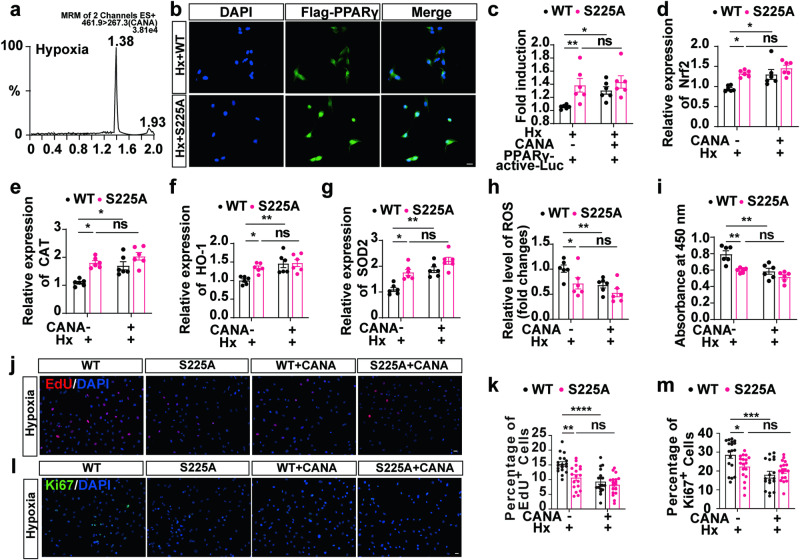
**CANA promotes the ability of anti-oxidative stress and anti-proliferation of PPARγ by inhibiting PPARγ S225 phosphorylation.** **a** Ultra-performance liquid chromatography chromatograms. **b** Immunostaining of Flag-PPARγ (green) and DAPI (blue) in PPARγ pcDNA3.1-3xFlag-C (WT) group and PPARγ S225A pcDNA3.1-3xFlag-C (S225A-PPARγ) group under hypoxia exposure. **c** Luciferase activity was determined using dual luciferase reporter system, (*n* = 6 per group from six independent experiments). qPCR analysis of NRF2 (**d**), CAT (**e**), HO-1 (**f**), and SOD2 (**g**) expression in WT, WT+CANA, S225A, S225A+CANA groups under hypoxia exposure (*n* = 6 per group from six independent experiments). **h** Intracellular ROS reacted with DCFH-DA and highly fluorescent DCF was detected by flow cytometry. Quantification analysis of DCF fluorescence on cells in WT, WT+CANA, S225A, S225A+CANA groups under hypoxia exposure (*n* = 6 per group from six independent experiments). **i** The fold changes of the CCK-8 result differences in the effects of CANA under WT or S225 treatments were analyzed (*n* = 6 per group from six independent experiments). Representative images of EdU staining of each group (**j**) and quantification analysis of EdU^+^ cells (**k**) in each group (*n* = 6 sections from six independent experiments, three images per section). Representative images of Ki67 positive cell staining of each group (**l**) and quantification analysis of Ki67^+^ cells (**m**) in each group (*n* = 6 sections from six independent experiments, three images per section), ^ns^*P* > 0.05, *P < 0.05, **P < 0.01, ***P < 0.001, ****P < 0.0001. Scale bars = 20 μm.

Liquid chromatography revealed that CANA was successfully taken up by rPASMCs, suggesting that CANA can enter cells and bind to PPARγ (Fig. 9a). We then generated and verified plasmids containing wild-type PPARγ (WT-PPARγ) and S225A mutant PPARγ (S225A-PPARγ, a nonphosphorylatable mutation, namely, serine was mutated to alanine) (Supplementary Fig. S7a, b, Supplementary Table 1). The silencing of PPARγ S225 site promoted the expression of PPARγ (Fig. S7c). Interestingly, compared with administration of WT-PPARγ, administration of S225A-PPARγ in rPASMCs resulted in an increase in nuclear translocation under hypoxia exposure (Fig. 9b). To further elucidate the effects of CANA and S225 phosphorylation on the transcriptional activation activity of PPARγ, we used a PPARγ-active-luc reporter system. Compared with WT-PPARγ, S225A-PPARγ promoted the transcriptional activity of PPARγ under hypoxia (Fig. 9c). In addition, compared to the non-CANA treatment, CANA promoted the transcriptional activity of PPARγ when coincubated with WT-PPARγ, but this promotion was not significant after coincubation with S225A-PPARγ (Fig. 9c), which indicated that the regulation of PPARγ transcriptional activity by CANA may be partially achieved by the inhibition of PPARγ phosphorylation at the S225 site.

We further investigated the effects of S225A-PPARγ on PPARγ-mediated transcriptional regulation associated with the oxidative stress response in rPASMCs. Compared with WT-PPARγ, S225A-PPARγ increased the expression of SOD2, CAT, NRF-2 and HO-1 (Fig. 9d–g) under hypoxia. CANA promoted the expression of SOD2, CAT, NRF-2 and HO-1 when incubated with WT-PPARγ, while this effect was almost eliminated after incubation with S225A-PPARγ (Fig. 9d–g). Similar to CANA treatment, S225A-PPARγ treatment decreased ROS accumulation in rPASMCs (Fig. 9h). When rPASMCs were transfected with S225A-PPARγ, the inhibition of oxidative stress by CANA was largely weakened compared with that in the non-CANA group, suggesting that CANA functions partially by inhibiting S225 phosphorylation. Additionally, the results of the CCK-8 assay (Fig. 9i), the EdU (Fig. 9j, k), and Ki67 (Fig. 9l, m) staining assays showed that the hypoxia-induced proliferation of the rPASMCs treated with S225A-PPARγ was decreased compared with that of the cells treated with WT-PPARγ, while there were no significant differences between the S225A-PPARγ and CANA groups and the S225A-PPARγ and non-CANA groups. Interestingly, CANA exhibited full antiproliferative effects when coincubated with WT-PPARγ, but this ability was markedly weakened when incubated with S225A-PPARγ (Fig. 9i–m).

Taken together, the data mentioned above indicate that CANA binds to PPARγ, preventing its S225 phosphorylation and subsequently promoting the nuclear translocation of PPARγ, thus alleviating PPARγ-mediated oxidative stress and proliferative inhibition.

## Discussion

PH, a life-threatening illness, is characterized by rPASMC proliferation and oxidative stress [40]. Although our understanding of the pathophysiology of PH has also advanced [41], there are still no effective drugs available to reverse the progression of PH. Recent clinical findings have indicated that CANA has an effect on cardiovascular disease [42]. However, the pharmacological effect of CANA on PH is currently unknown. In this study, we propose that CANA can be utilized as a therapeutic agent for PH treatment. This hypothesis was supported by the following evidence: (1) CANA reduced the elevated pressure in the pulmonary artery, hypertrophy of the right ventricle, remodeling of the pulmonary blood vessels, and levels of oxidative stress in the pulmonary vessels in vivo; (2) the excessive proliferation and migration of hypoxia-induced rPASMCs were relieved by CANA treatment; (3) by upregulating PPARγ, CANA inhibited excessive oxidative stress, thereby mitigating rPASMC proliferation; and (4) CANA suppressed the phosphorylation of PPARγ S225, facilitating PPARγ nuclear translocation and increasing PPARγ functionality.

This study utilized network pharmacology and transcriptomics to investigate the therapeutic mechanism of CANA in PH, followed by experimental validation. Network pharmacology, an interdisciplinary field that combines biology, chemical informatics, and traditional bioinformatics pharmacology, helps scientists manage increasing amounts of data [43, 44]. First, the targets of CANA were screened by the reverse docking procedure, and the targets of PH were determined from multiple databases. Among the 25 CANA-anti-PH targets, PPARγ, MMP9, iNOS, eNOS, and ACE were centrally located in the PPI network. iNOS and eNOS, which are crucial enzymes in the synthesis of NO, have a major impact on the preservation of vascular endothelial function [45, 46]. MMP9 can participate in extracellular matrix turnover, promoting rPASMC proliferation and migration and leading to pulmonary arterial remodeling [47]. ACE also promoted rPASMC proliferation and migration, which contributed to the pathogenesis of PH [48]. PPARγ was detected on smooth muscle cells and endothelial cells in the pulmonary vasculature [49], and its activation was strongly associated with cell growth and the presence of oxidative stress [50]. Additionally, PPARγ plays a crucial role in regulating oxidative stress by modulating various pathways involved in antioxidant defense mechanisms. Activation of PPARγ has been shown to enhance the expression of antioxidant enzymes such as superoxide dismutase (SOD) and catalase [51–53], thereby reducing ROS levels and mitigating oxidative damage. Additionally, PPARγ activation can suppress the proliferation of vascular smooth muscle cells by inhibiting oxidative stress [54].

The protein expression of PPARγ in the lung tissues of PH patients and PH model mice was found to be significantly decreased, as shown by our group and other research teams [27, 55]. In PH mouse models, increased expression of PPARγ effectively reversed the remodeling of pulmonary blood vessels and relieved the symptoms of PH [28, 56]. PPARγ activation also suppressed rPASMC proliferation and promoted eNOS expression, increasing the release of NO from endothelial cells [57, 58]. The results of this study indicate that CANA significantly increases the expression of PPARγ both in vivo and in vitro. Based on the evidence above, we presumed that PPARγ plays a key role in the anti-PH effects of CANA. The other 4 core genes (eNOS, iNOS, ACE, MMP9) were not initially considered for the following reasons: (1) our transcriptome data indicated significant enrichment of the PPAR signaling pathway after CANA treatment, highlighting the importance of PPARγ in the anti-PH effect of CANA; (2) PPARγ showed a strong association with oxidative stress, which was also significantly enriched in the transcriptome and network pharmacology analysis; and (3) our previous studies emphasized the crucial role of PPARγ in PH pathology in both rPASMCs and endothelial cells, while eNOS, iNOS, and ACE were primarily associated with endothelial cells [45, 46, 59], and MMP9 was related to fibroblasts [60, 61]. Since PAMSCs are key cells involved in vascular remodeling, our study focused specifically on the role of rPASMCs in the pathogenesis of PH. However, further research is needed to determine whether CANA affects lung endothelial cells through PPARγ.

CANA, dapagliflozin, and empagliflozin are classified as inhibitors of sodium-glucose cotransporter 2 (SGLT2), where SGLT2 accounts for the majority of renal glucose reabsorption in the kidneys [62]. Various studies have shown that CANA has a weaker inhibitory effect on SGLT2 than dapagliflozin and empagliflozin, suggesting that CANA might exhibit more specific and potent inhibition of other proteins [63, 64]. Similar to scholarly research indicating the absence of SGLT2 in any blood vessel other than the kidney, our investigation using the THPA database revealed a low level of SGLT2 protein in rPASMCs. Hence, we did not examine the effect of CANA on SGLT2 in our models. Our findings confirmed that CANA can directly bind to PPARγ, thereby hindering its phosphorylation. This discovery introduces a novel perspective on CANA. Notably, in addition to SGLT2 inhibitors regulating blood sugar levels, SGLT2 inhibitors such as CANA are being increasingly used due to their supplementary advantages for cardiovascular health [65, 66].

In PH associated with heart failure with preserved ejection fraction (HFpEF), Taijyu et al. [67] reported that empagliflozin decreased mitochondrial ROS and increased pulmonary arterial remodeling while exercising. ZSF-1 obese rats were used as a rodent model for HFpEF and PH, either with or without Sugen treatment. These models exhibit numerous characteristics similar to those of type II diabetes in humans [68]. According to the authors, in this particular situation, empagliflozin ameliorated metabolic syndrome, thereby inhibiting the initial development of mitochondrial ROS [67]. We utilized hypoxia-related pulmonary hypertension (PH) models from the third category, which lacked a distinct metabolic profile (such as diabetes) similar to that of obese ZSF-1 rats. Hence, these metabolic features were not evaluated in this study. Similar to the role of empagliflozin in HFpEF, CANA also ameliorated oxidative stress and pulmonary artery remodeling in hypoxia-related PH in our study. However, we highlight the role of PPARγ in the anti-PH effects of CANA. Indeed, PPARγ and the PPAR coactivator PGC1-α are thought to be closely related to mitochondrial oxidative stress. In addition, there is no direct evidence that CANA modulates oxidative stress via SLGT2 inhibition.

In short, different SGLT2 inhibitors might have different effects on different clinical types of PH. Hess et al. [69] found that empagliflozin attenuated MCT-induced PH, but its molecular mechanism of action was nonspecific. Wu et al. [70] showed that dapagliflozin has no protective effect on PH models. Recently, Tang et al. [71] showed that CANA had a protective effect on hypoxia-induced PH, which is consistent with our conclusions. However, our exploration of the role and mechanism of CANA in PH is undoubtedly multiangled, comprehensive, and in depth.

PPARγ activation has been found to prevent PH progression. According to our previous investigation, rosiglitazone, a conventional activator of PPARγ, can effectively alleviate pulmonary vascular remodeling and right ventricular remodeling in a mouse model of hypoxia-induced PH [28]. Legchenko et al. reported that the PPARγ agonist pioglitazone reversed PH and prevented right heart failure [52]. Pioglitazone was also found to prevent the development of MCT-induced PH in rats [72]. However, several clinical trials with these PPARγ agonists, such as rosiglitazone, have demonstrated that they may increase heart failure. This finding can be explained by their side effects, with a focus on mechanisms such as edema and sodium retention. The clinical use of full-spectrum PPARγ agonists, such as rosiglitazone, is limited due to the cardiovascular risks associated with their long-term use. In the treatment of diabetes, one strategy is the development of selective PPARγ modulators, SPPARMs, to preserve the expression level of PPARγ in vivo and regulate the transcription of therapeutic-related target genes while avoiding the regulation of target genes linked to adverse reactions [73]. Our data provide some support for the use of CANA as a selective PPARγ modulator, which holds promise for the treatment of PH. However, more experiments are needed to support this hypothesis.

Protein functions can be regulated by post-translational modifications, most commonly phosphorylation [74]. Depending on the cell type and stimulation, the site of phosphorylation of PPARγ and its biological effects are completely different [75, 76]. The phosphorylation sites that have been identified in PPARγ include Ser112 and Ser273. PPARγ Ser112 phosphorylation typically leads to reduced PPARγ activity [77], while PPARγ phosphorylation at Ser273 occurs mainly in adipose tissue [78]. Phosphorylation of PPARγ Ser225 (S225) has not been reported. Our results suggested that PPARγ S225 expression was altered in the pathogenesis of PH, which inhibited PPARγ transfer into the nucleus and thus reduced PPARγ transcriptional activity. However, CANA directly interacts with PPARγ, inhibiting PPARγ S225 phosphorylation and promoting PPARγ activity, resulting in excellent antioxidant stress effects. However, a larger in vivo dataset would result in further insight into this issue.

The findings of this research suggest that CANA has potential for use as a treatment for PH. This drug improved the progression of PH by reducing damage caused by oxidative stress and inhibiting the proliferation of smooth muscle cells in the pulmonary artery. This effect is partially achieved by activating PPARγ. Mechanistically, CANA inhibits PPARγ S225 phosphorylation and promotes PPARγ function. Taken together, the results of this study may help to elucidate the molecular process underlying the emergence and progression of PH while also revealing a novel avenue for the practical utilization of CANA.

### Supplementary information

Supplementary Fig. S1

Supplementary Fig. S2

Supplementary Fig. S3

Supplementary Fig. S4

Supplementary Fig. S5

Supplementary Fig. S6

Supplementary Fig. S7

Supplementary Table 1

Supplementary Table 2

Raw data for WB

## Data Availability

Data will be made available upon request.
