# Supplementary figures and images for: Canagliflozin alleviates pulmonary hypertension by activating PPARγ and inhibiting its S225 phosphorylation
Source: Acta Pharmacol Sin. 2024 May 8;45(9):1861–78. doi: 10.1038/s41401-024-01286-9 (PMC11335861; doi:10.1038/s41401-024-01286-9)

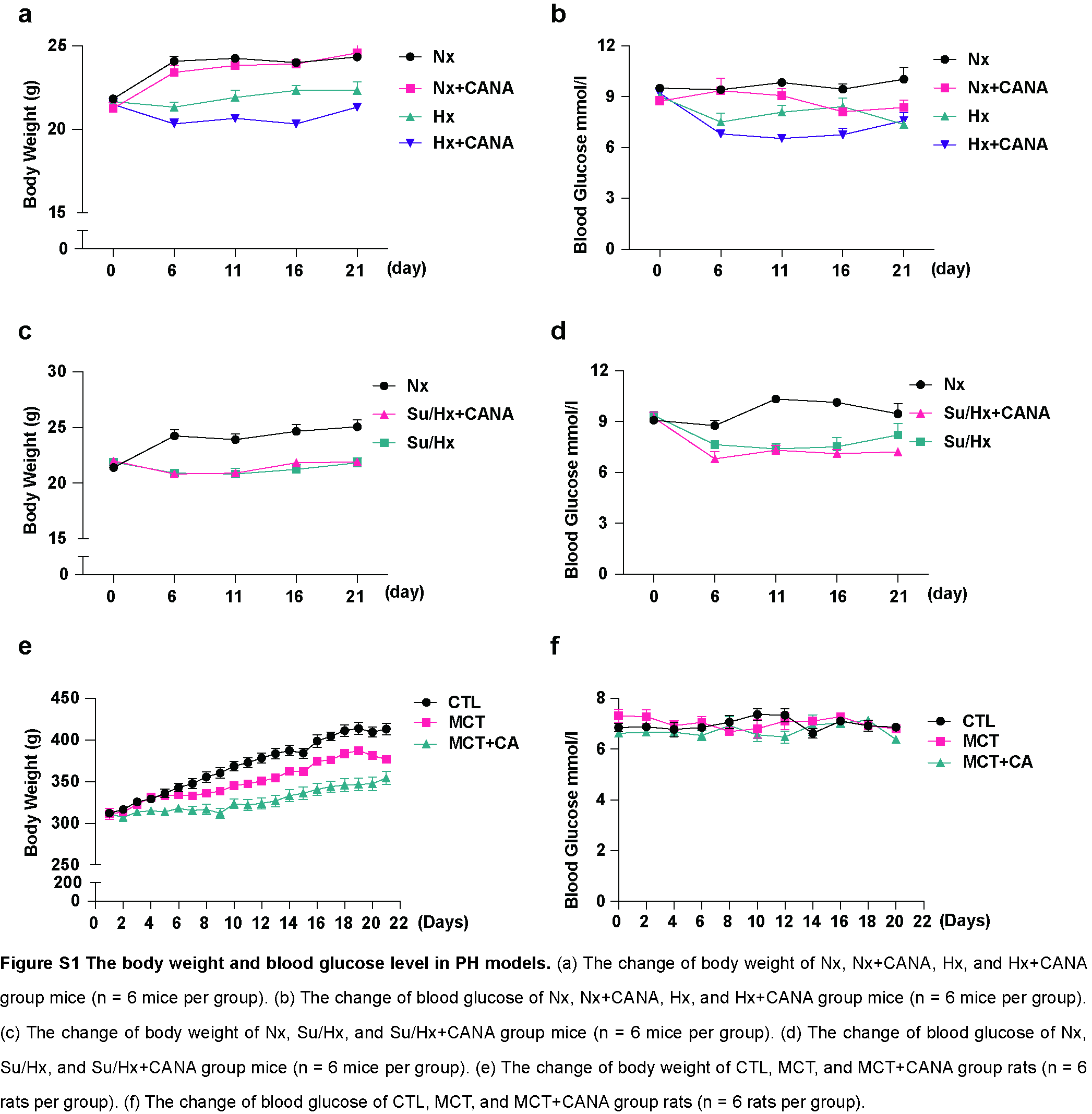

Supplement: Supplementary file 1 — Supplementary Fig. S1 [file 41401_2024_1286_MOESM1_ESM.tif]

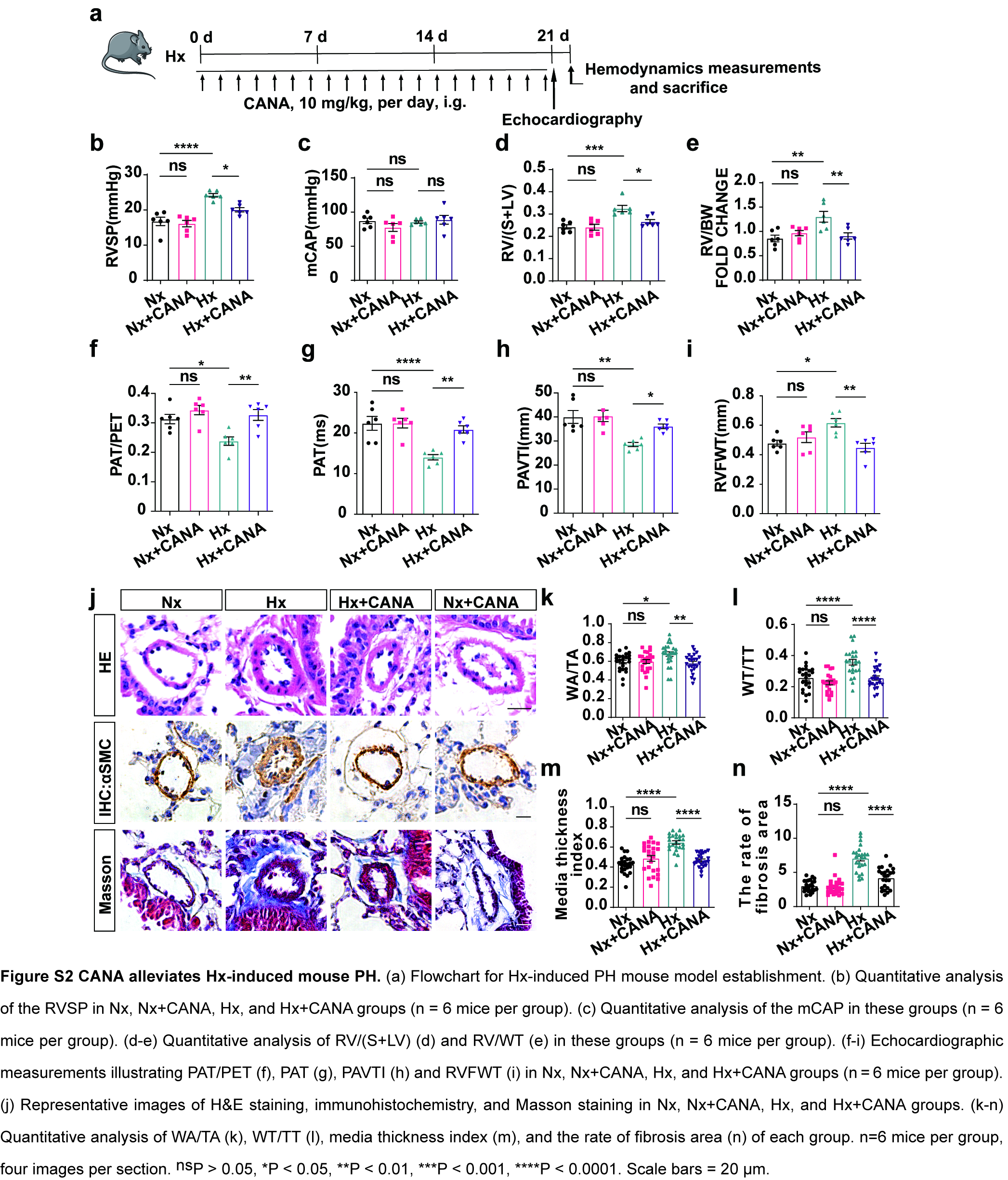

Supplement: Supplementary file 2 — Supplementary Fig. S2 [file 41401_2024_1286_MOESM2_ESM.tif]

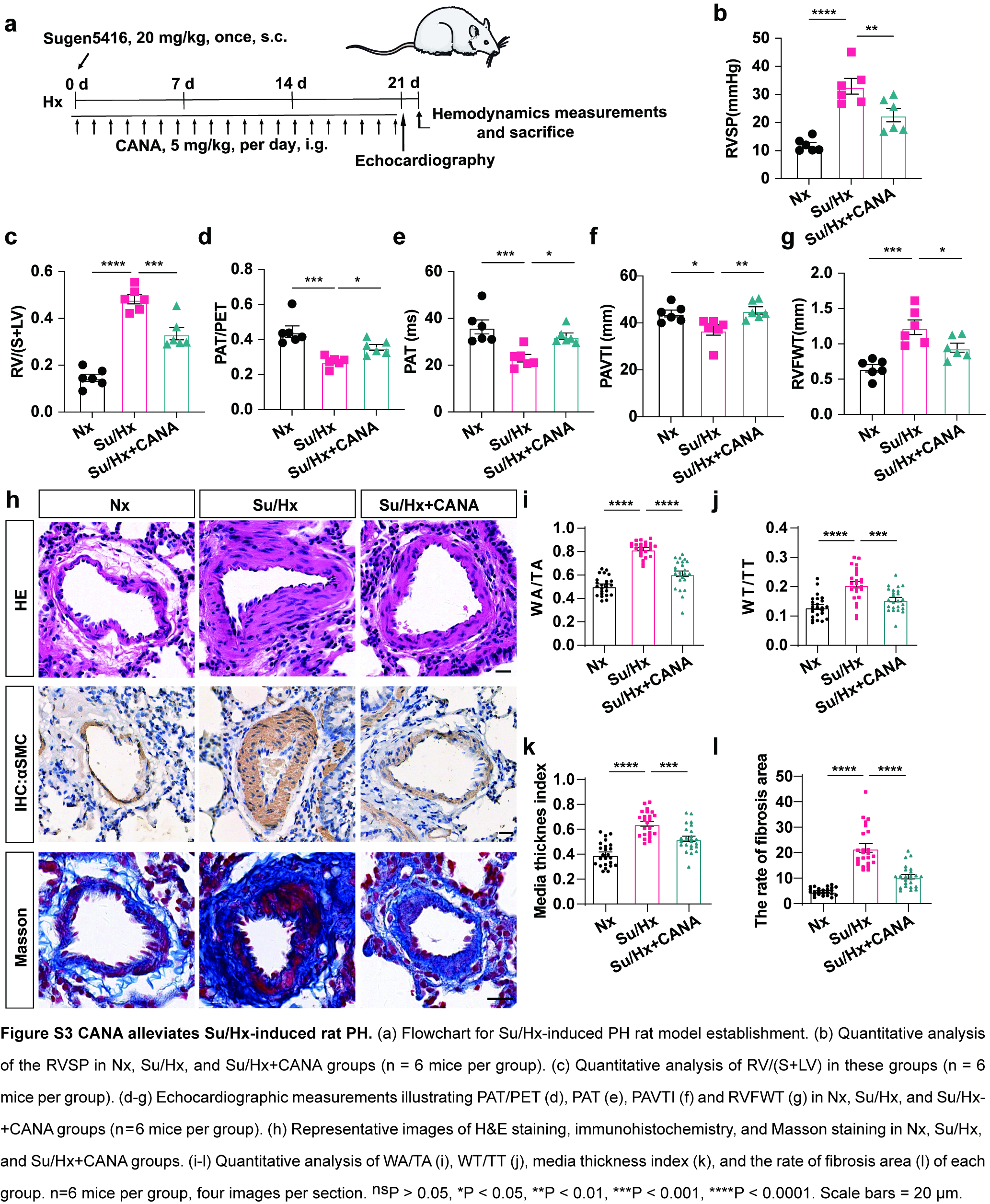

Supplement: Supplementary file 3 — Supplementary Fig. S3 [file 41401_2024_1286_MOESM3_ESM.tif]

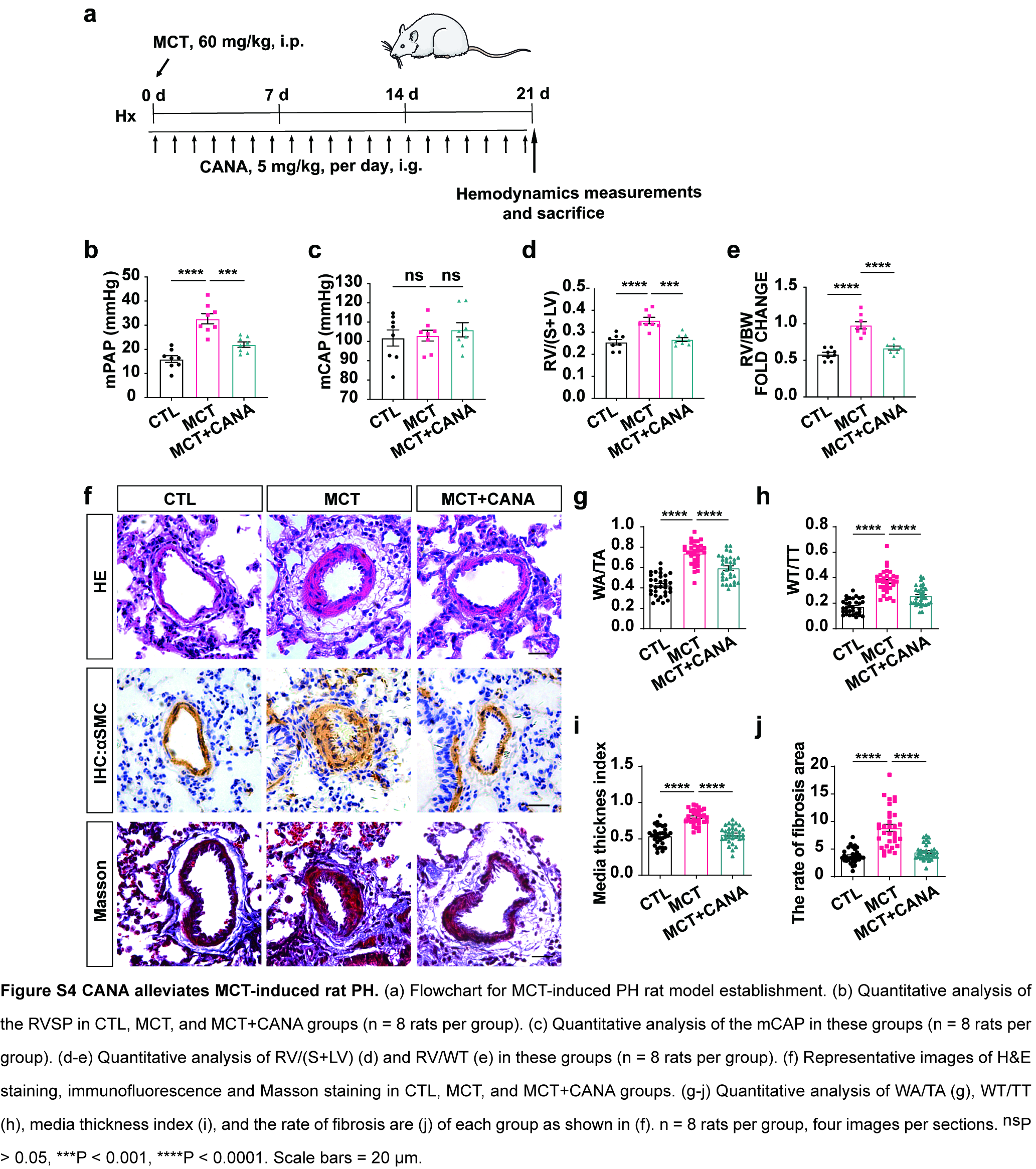

Supplement: Supplementary file 4 — Supplementary Fig. S4 [file 41401_2024_1286_MOESM4_ESM.tif]

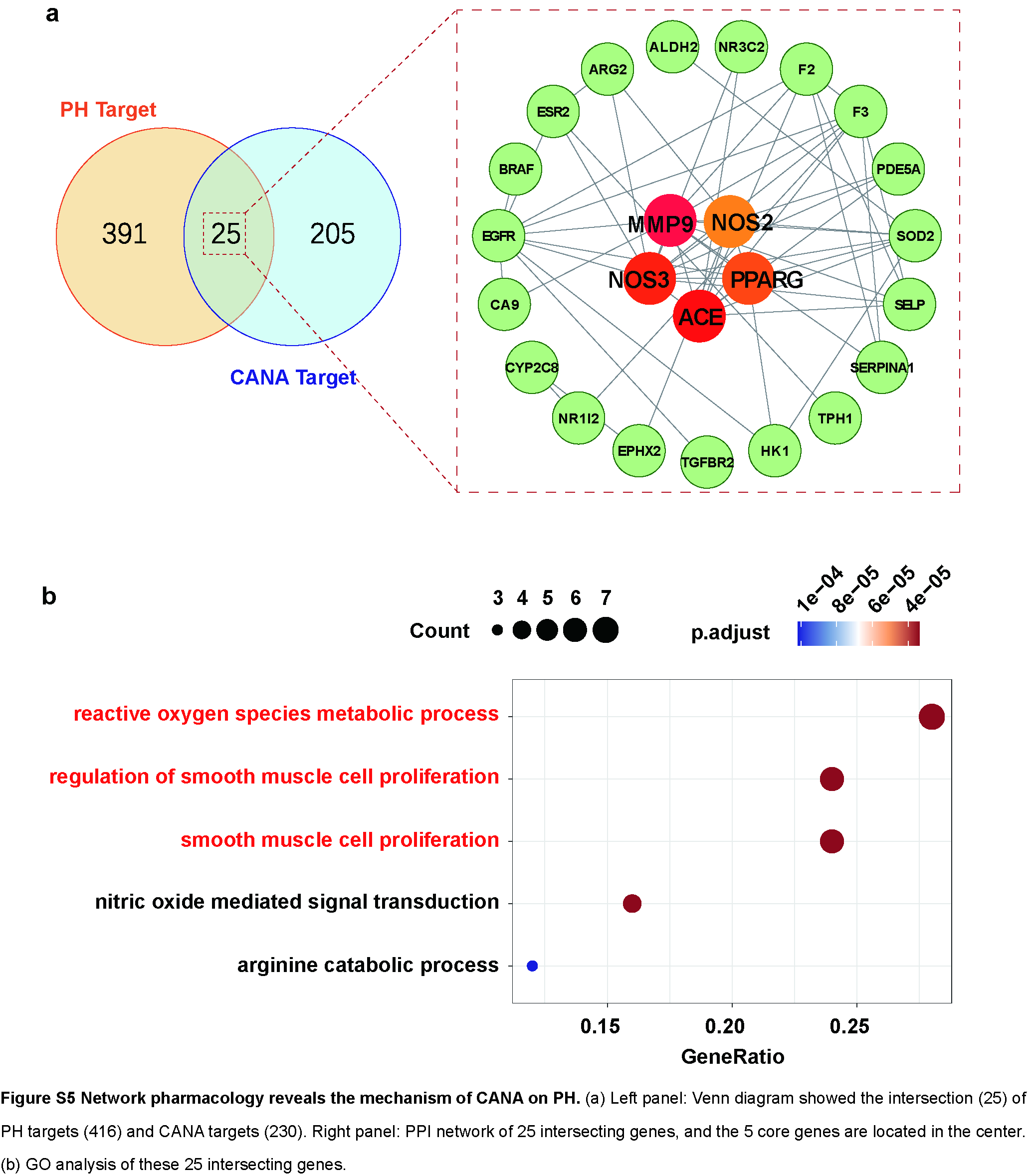

Supplement: Supplementary file 5 — Supplementary Fig. S5 [file 41401_2024_1286_MOESM5_ESM.tif]

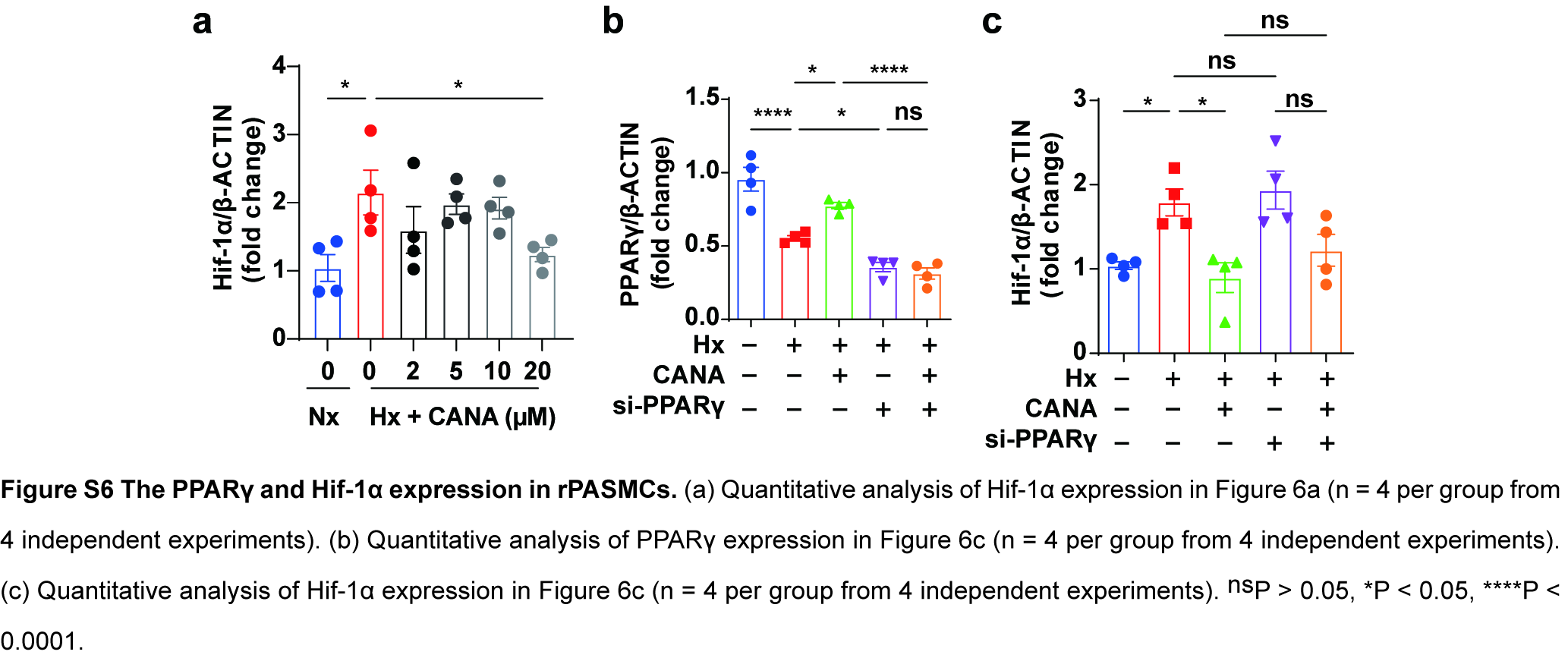

Supplement: Supplementary file 6 — Supplementary Fig. S6 [file 41401_2024_1286_MOESM6_ESM.tif]

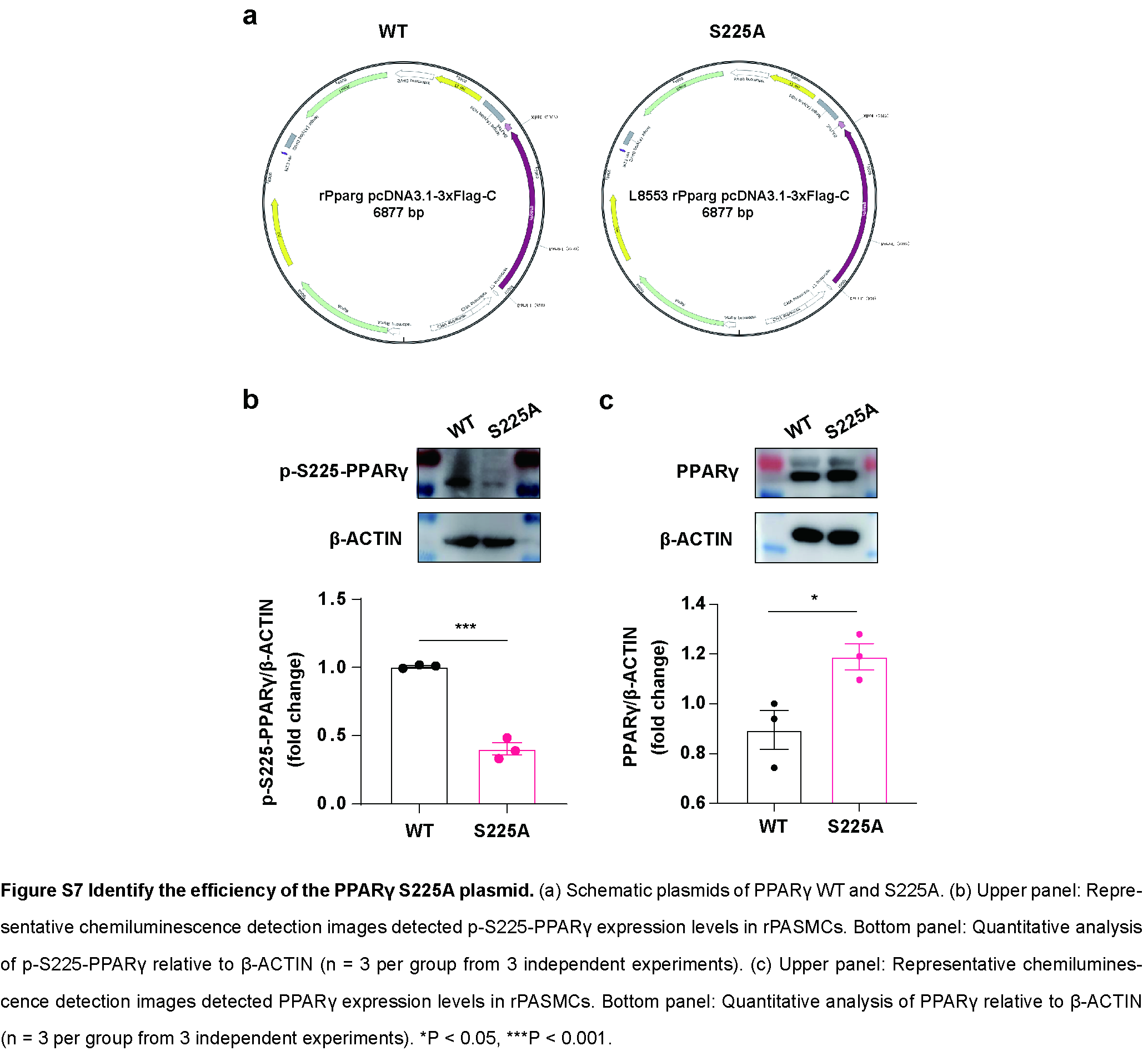

Supplement: Supplementary file 7 — Supplementary Fig. S7 [file 41401_2024_1286_MOESM7_ESM.tif]

## Slide 1
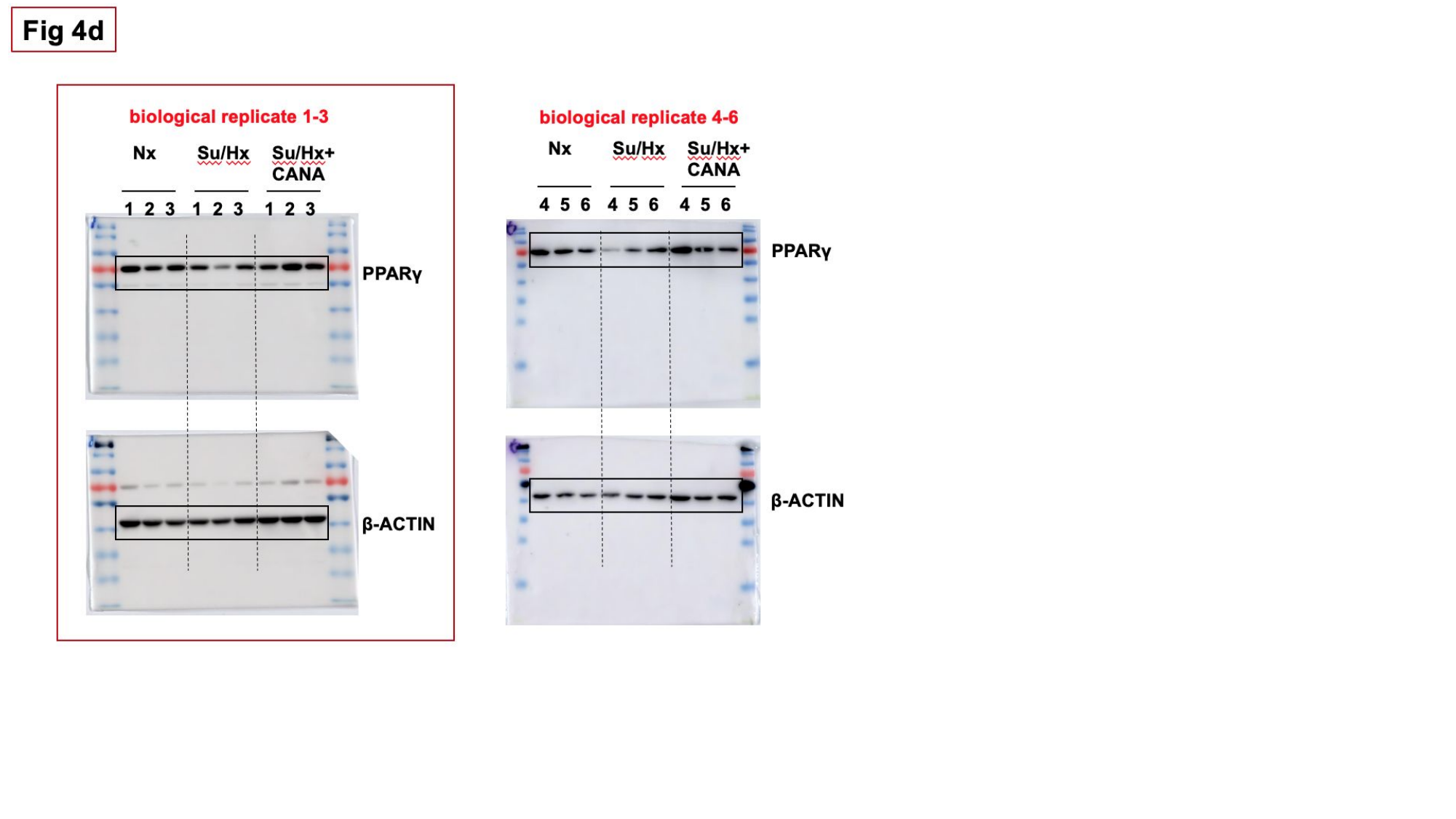

## Slide 2
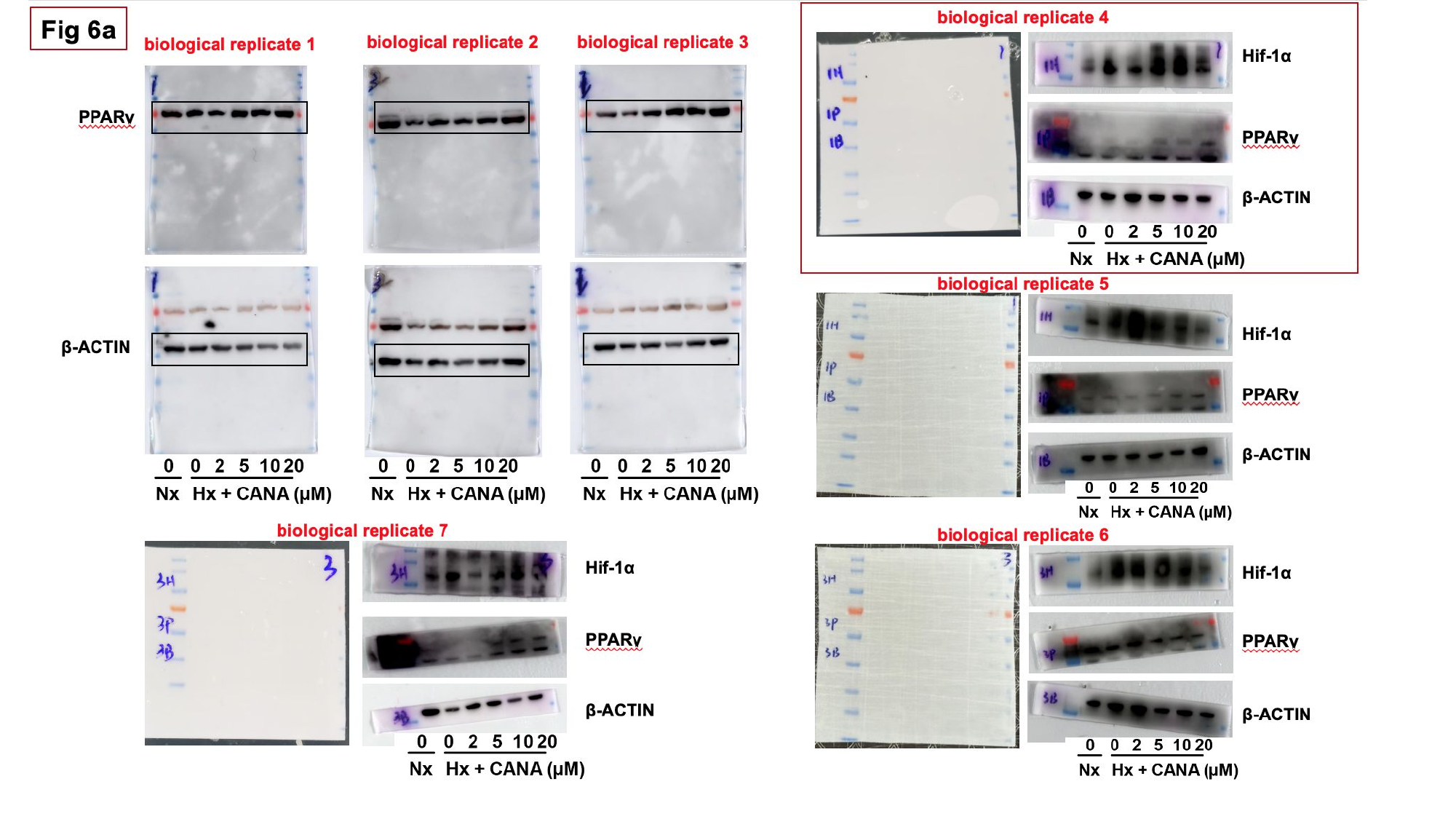

## Slide 3
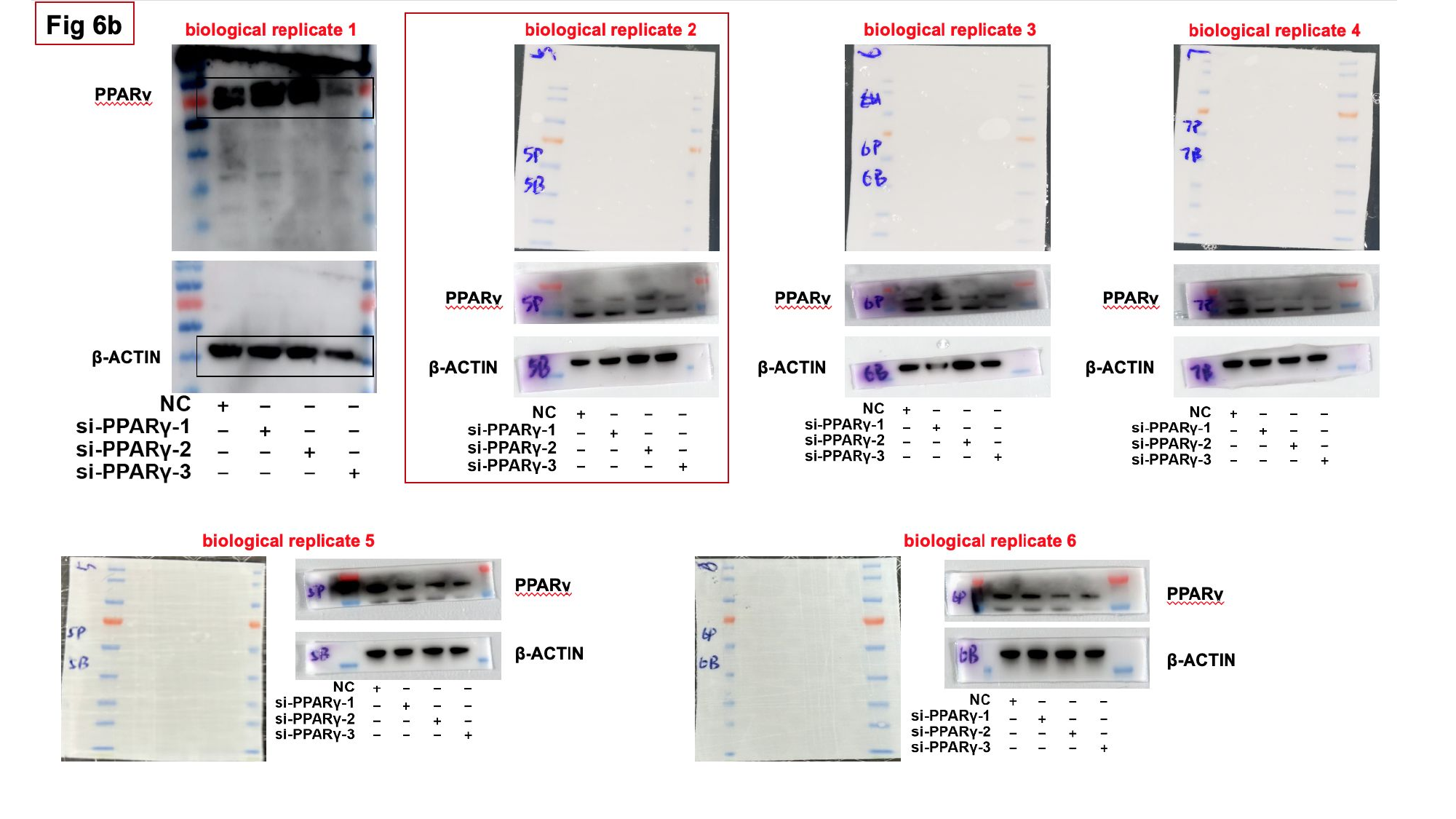

## Slide 4
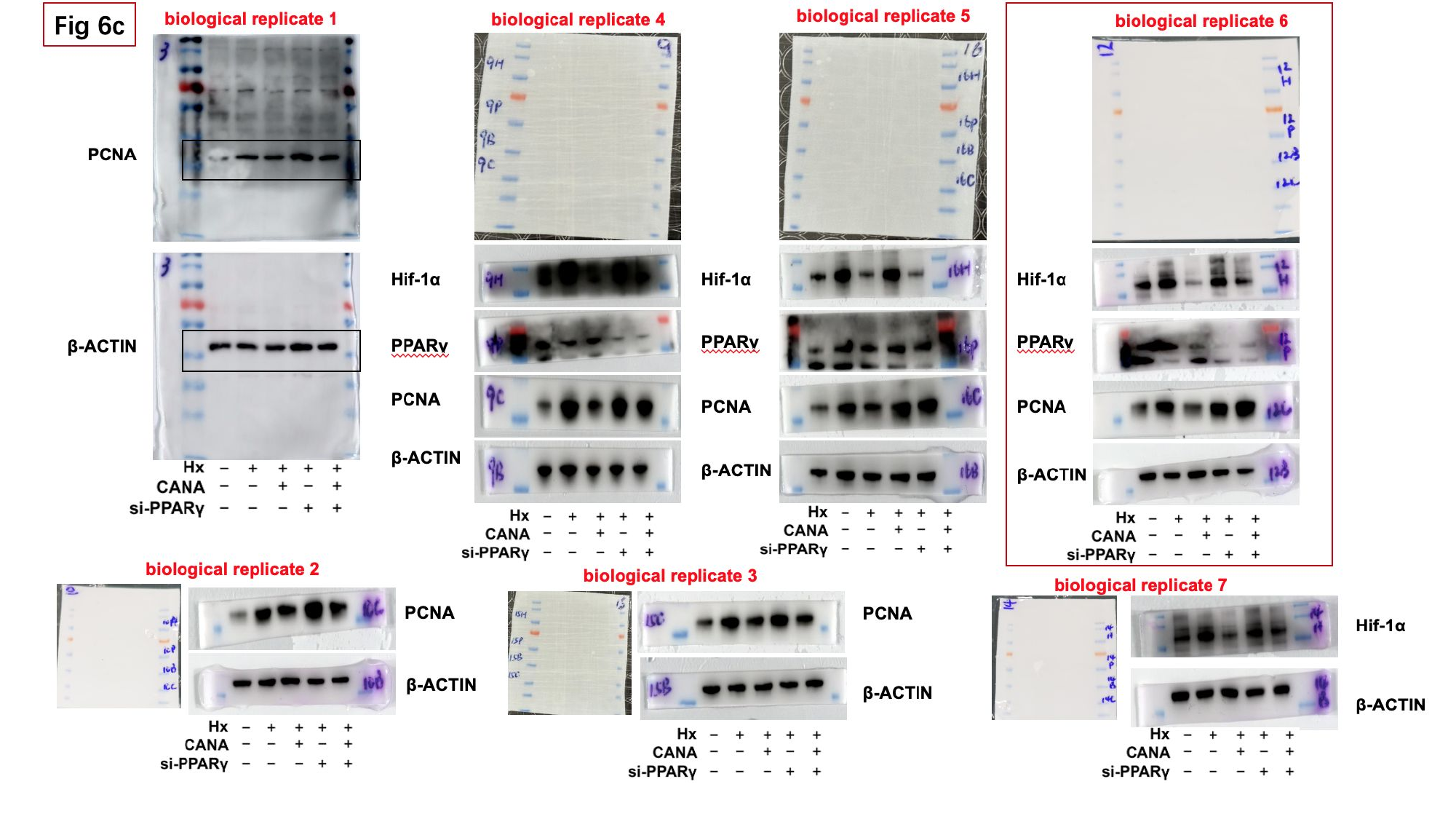

## Slide 5
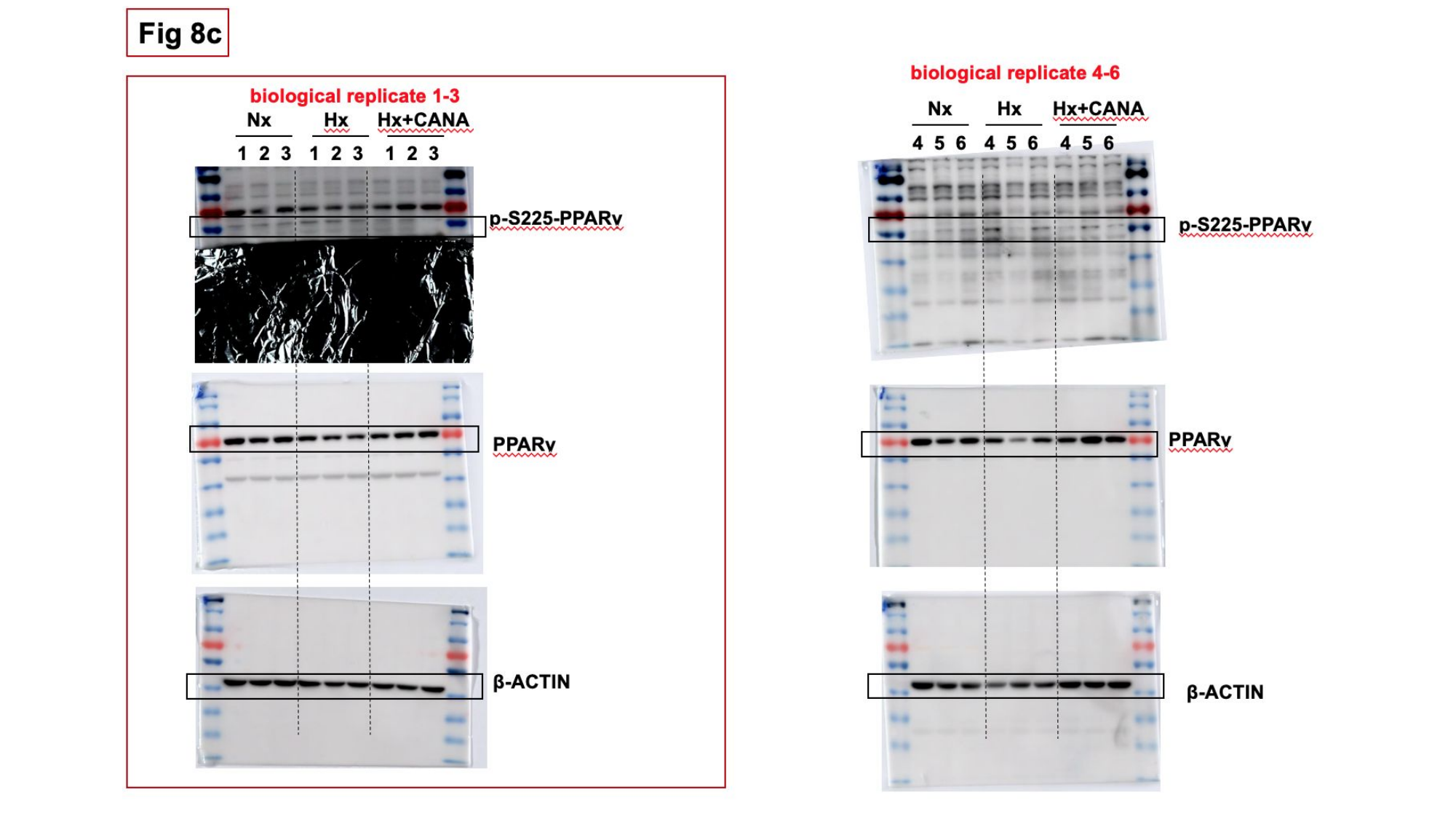

## Slide 6
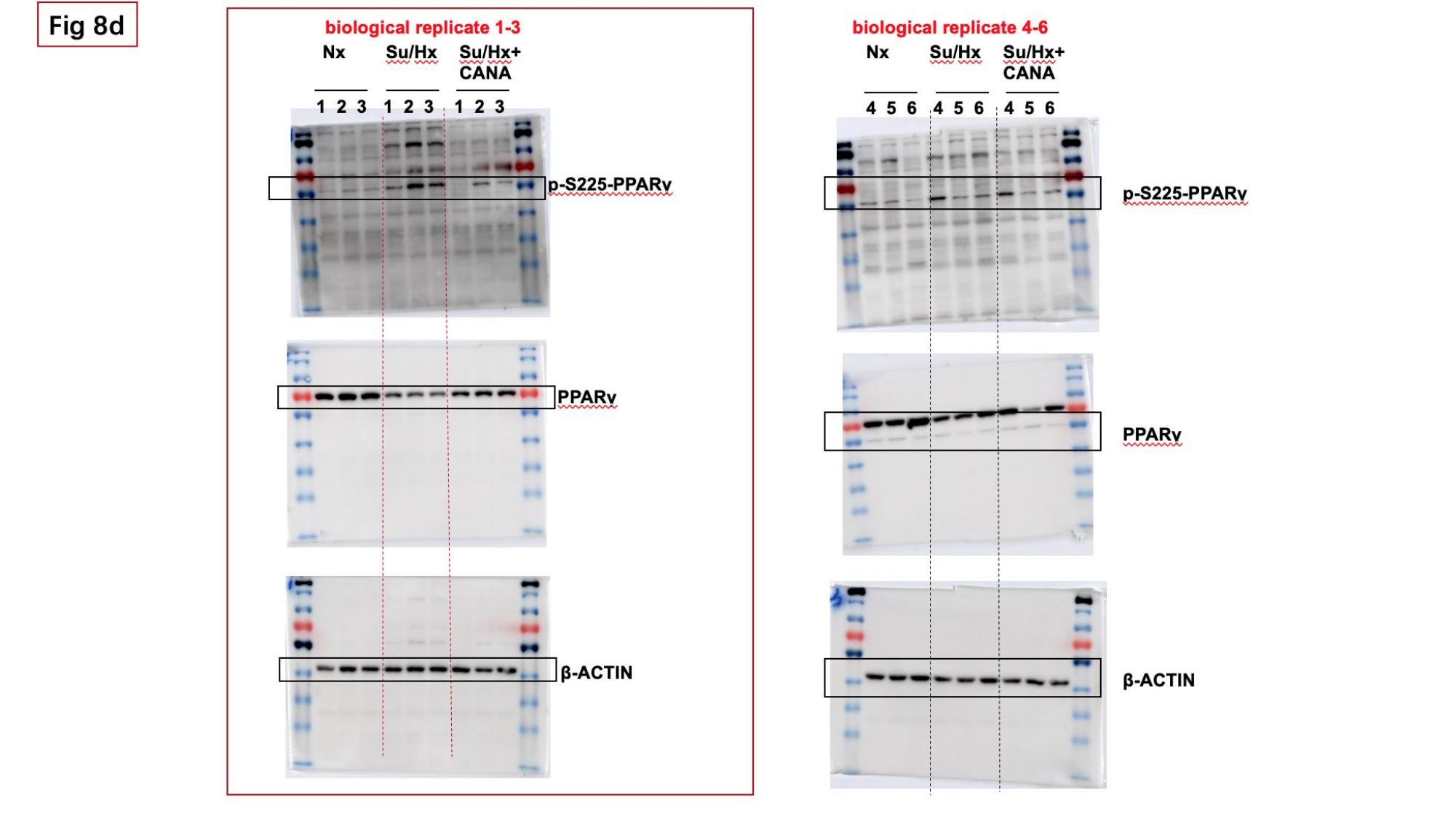

## Slide 7
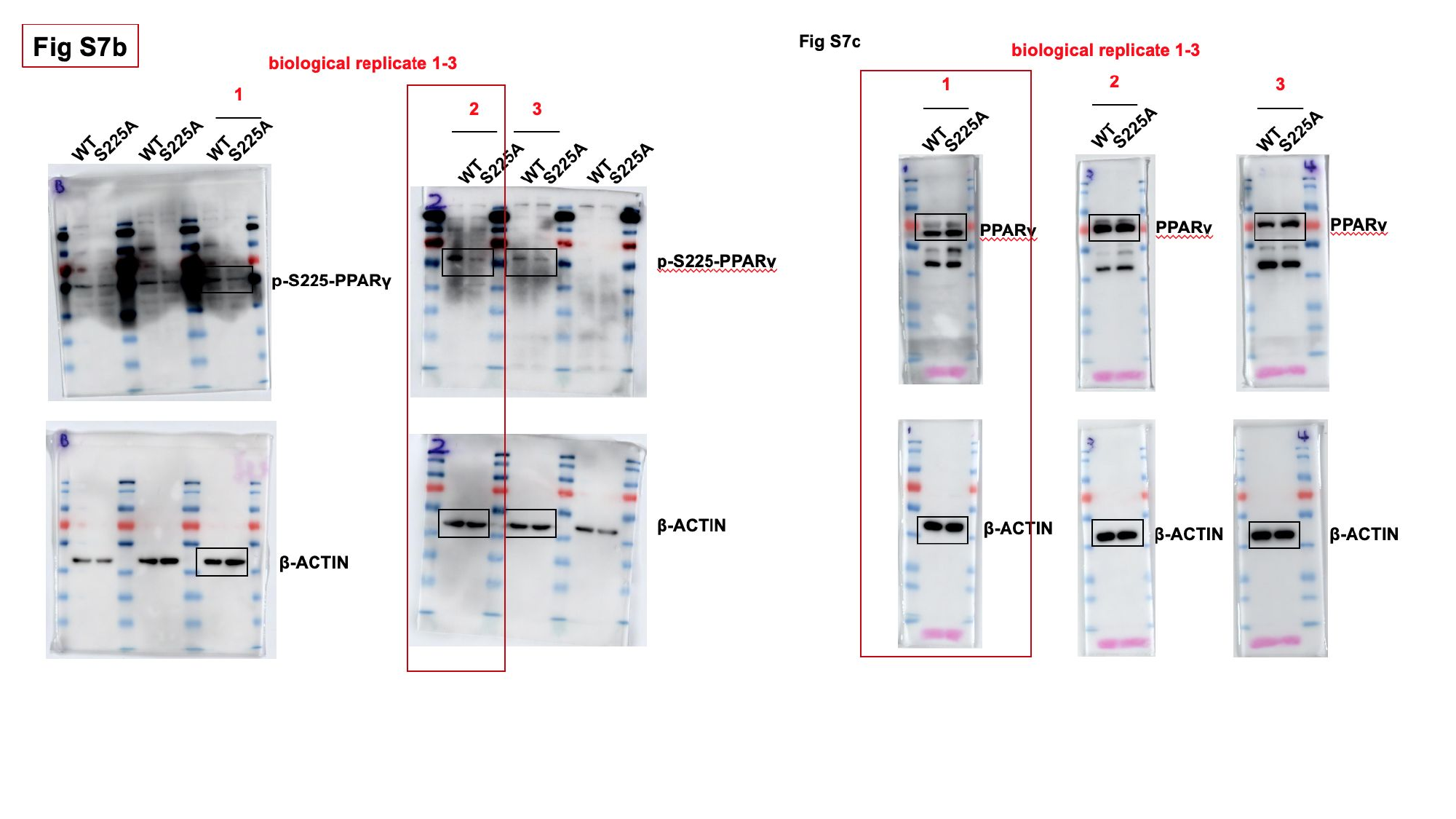

Supplement: Supplementary file 10 — Raw data for WB [file 41401_2024_1286_MOESM10_ESM.pptx]
