# Supplementary material for: Canagliflozin alleviates pulmonary hypertension by activating PPARγ and inhibiting its S225 phosphorylation
Source: Acta Pharmacol Sin. 2024 May 8;45(9):1861–78. doi: 10.1038/s41401-024-01286-9 (PMC11335861; doi:10.1038/s41401-024-01286-9)
Supplement: Supplementary file 8 — Supplementary Table 1 [file 41401_2024_1286_MOESM8_ESM.docx]

**Supplementary Table 1. The antibodies used in this study.**

| **Antibody** | **Source** | **Identifier** | **ratio** | **application** |
| --- | --- | --- | --- | --- |
| αSMC (RRID: AB_262054) | Santa | sc-32251 | 1:400 | IHC |
| PPARγ | Abcam | ab272718 | 1:1000 | WB |
| HIF-1α | CST | #14179 | 1:1000 | WB |
| PCNA | CST | #13110 | 1:1000 | WB |
| β-ACTIN | CST | #4970 | 1:1000 | WB |
| p-S225-PPARγ | HuaAn bio | / | 1:1000 | WB |
| Ki67 (RRID: AB_302459) | Abcam | ab16667 | 1:250 | IF |
| Flag (RRID: AB_2916341) | Abcam | ab205606 | 1:250 | IF |
| goat anti-rabbit IgG (RRID: AB_2819160) | Abcam | ab205718 | 1:10000 | WB |
| Donkey anti-Rabbit IgG (H+L) Highly Cross-Adsorbed Secondary Antibody, Alexa Fluor™ 488 | Invitrogen | A21206 | 1:1000 | IF |
